# Supplementary material for: Ultrafast decoupling of polarization and strain in ferroelectric BaTiO3
Source: Nat Commun. 2025 Aug 26;16:7966. doi: 10.1038/s41467-025-63045-6 (PMC12381263; doi:10.1038/s41467-025-63045-6)
Supplement: Supplementary file 1 — Supplementary Information [file 41467_2025_63045_MOESM1_ESM.pdf]

Supplementary Information for: Ultrafast decoupling of polarization and strain in ferroelectric $BaTiO_3$

Le Phuong Hoang[1,2,3], David Pesquera[4], Gerard N. Hinsley[5], Robert Carley[1], Laurent Mercadier[1], Martin Teichmann[1], Elena Martina Unterleutner[6], Daniel Knez[6], Martina Dienstleder[7], Saptam Ganguly[4], Teguh Citra Asmara[1], Giacomo Merzoni[8,1], Sergii Parchenko[1], Justine Schlappa[1], Zhong Yin[1,13], José Manuel Caicedo Roque[4], José Santiso[4], Irena Spasojevic[9], Cammille Carinan[1], Tien-Lin Lee[10], Kai Rossnagel[3,11], Jörg Zegenhagen[10], Gustau Catalan[4,12], Ivan A. Vartanyants[5], Andreas Scherz[1], and Giuseppe Mercurio[1,*]

[1]*European XFEL, Schenefeld, Germany*

[2]*Max Planck Institute for the Structure and Dynamics of Matter, Hamburg, Germany*

[3]*Institute of Experimental and Applied Physics, Kiel University, Kiel, Germany*

[4]*Catalan Institute of Nanoscience and Nanotechnology (ICN2), CSIC and BIST, Campus UAB, Bellaterra, Spain*

[5]*Photon Science, Deutsches Elektronen-Synchrotron DESY, Hamburg, Germany*

[6]*Institute of Electron Microscopy and Nanoanalysis (FELMI), Graz University of Technology, Graz, Austria*

[7]*Graz Centre for Electron Microscopy (ZFE), Graz, Austria*

[8]*Dipartimento di Fisica, Politecnico di Milano, Milano, Italy*

[9]*Department de Física, Universitat Autònoma de Barcelona, Bellaterra, Spain*

[10]*Diamond Light Source Ltd., Didcot, Oxfordshire, UK*

[11]*Ruprecht Haensel Laboratory, Deutsches Elektronen-Synchrotron DESY, Hamburg, Germany*

[12]*Institut Català de Recerca i Estudis Avançats (ICREA), Barcelona, Spain*

[13]*Present address: International Center for Synchrotron Radiation Innovation Smart (SRIS), Tohoku University, Sendai, Japan and*

[*]*Corresponding author: giuseppe.mercurio@xfel.eu*

Contents

Supplementary Figure 1: Diffraction intensity drop 5

Supplementary Figure 2: Contribution of Debye-Waller factor 6

Supplementary Table 1: Physical constants 7

Supplementary Note 1. Two-temperature model 8

Supplementary Figure 3: Electron and lattice temperature as a function of delay t 11

Supplementary Note 2. Strain model 12

Supplementary Figure 4: $\overline{\eta}(t)$ with pump fluence $F_{\text{in}} = 1.4$ mJ/cm^2 14

Supplementary Table 2: Strain model fit results 14

Supplementary Figure 5: Lattice temperature as a function of delay and depth 15

Supplementary Figure 6: Deformation potential and thermoelastic contributions 16

Supplementary Note 3. Estimation of the bandgap decrease 16

Supplementary Note 4. Temperature, strain and diffraction curve calculations 17

Supplementary Figure 7: Strain model calculations with $F_{\text{in}} = 2.7$ mJ/cm^2 19

Supplementary Figure 8: Strain model calculations with $F_{\text{in}} = 1.4$ mJ/cm^2 20

Supplementary Figure 9: Delay dependence of the tensor elements $\chi^{(2)}_{xxz}$, $\chi^{(2)}_{zxx}$, and $\chi^{(2)}_{zzz}$ 21

Supplementary Note 5. Fit function of tr-XRD, tr-SHG and tr-refl delay scans 22

Supplementary Figure 10: SHG and R maximum relative change 23

Supplementary Figure 11: SHG and R time traces at different pump fluences 24

Supplementary Figure 12: Photoexcited electron density as a function of delay and fluence 25

Supplementary Note 6. Estimation of the carrier diffusion length L_d 25

Supplementary Note 7. Sample properties 26

A Transmittance profile of 266 nm beam in BTO/SRO/GSO 26

Supplementary Figure 13: Transmittance profile 26

Supplementary Table 3: Absorbed fluences in BTO and SRO thin films 27

B Penetration depths 27

Supplementary Note 8. Estimation of the electronic contribution to the ferroelectric polarization magnitude P_s 28

Supplementary Figure 14: θ-2θ scan 29

Supplementary Figure 15: c parameters as a function of sample temperature 30

Supplementary Figure 16: Reciprocal space map 31

Supplementary Note 9. Scanning transmission electron microscopy 32

Supplementary Figure 17: STEM images and EDX elemental maps 33

Supplementary Figure 18: Piezoresponse force microscopy 34

Supplementary Figure 19: SHG plots at different azimuthal angles 35

Supplementary Figure 20: Comparison of $I_{\mathrm{XRD}}(E_\nu)$ at negative delay and laser off 36

Supplementary Figure 21: Determination of t_0 in tr-XRD experiments 37

Supplementary Note 10. Bunch arrival time monitor and time resolution of tr-XRD experiments 38

Supplementary Figure 22: Beam arrival monitor 39

Supplementary Figure 23: BTO, SRO and GSO (001) diffraction peaks 40

Supplementary Figure 24: Comparison of simulated and experimental $I_{\mathrm{XRD}}(E_\nu)$ 41

Supplementary Figure 25: SHG intensity as a function of probe pulse energy 42

References 43

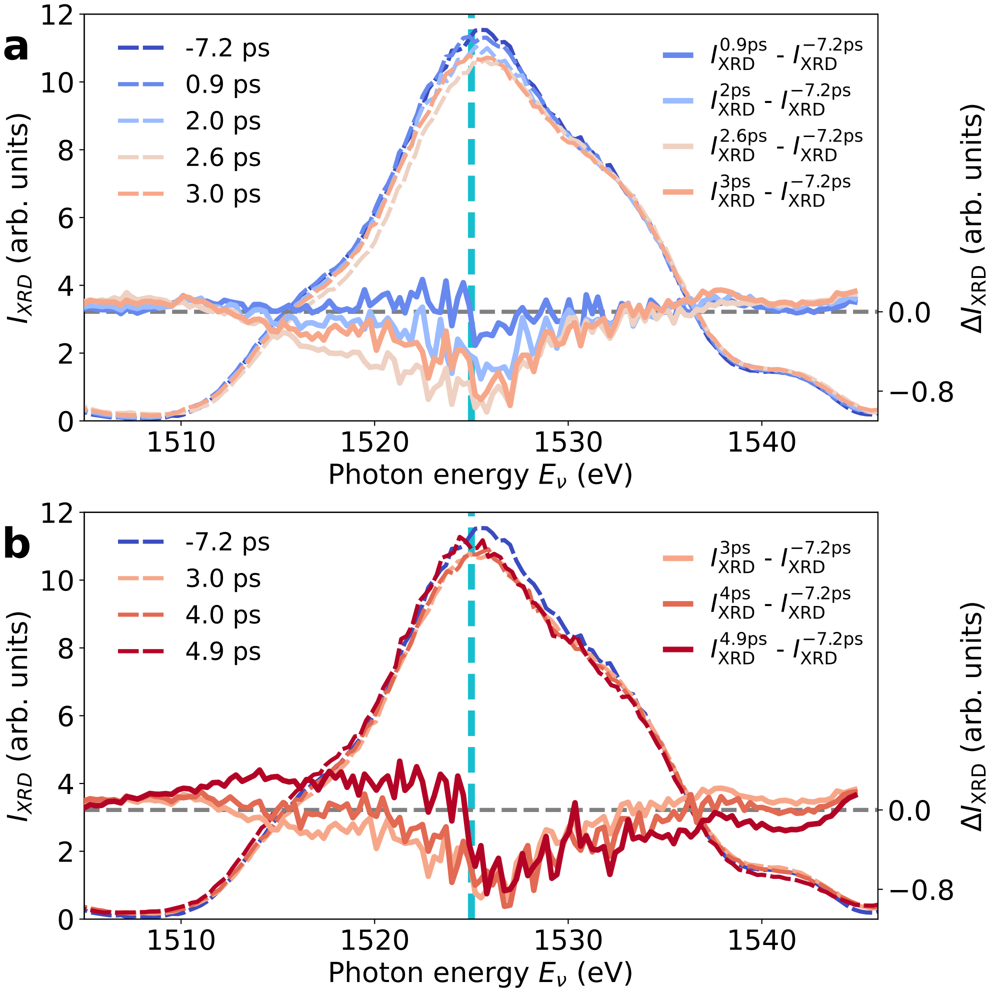

Supplementary Figure 1. **Diffraction intensity drop.** Experimental $I_{\mathrm{XRD}}(E_\nu)$ curves at different pump-probe delays t, and the respective differences $\Delta I_{\mathrm{XRD}} = I^t_{\mathrm{XRD}} - I^{-7.2\,\mathrm{ps}}_{\mathrm{XRD}}$, where $t = 0.9\,\mathrm{ps}, 2\,\mathrm{ps}, 2.6\,\mathrm{ps}, 3\,\mathrm{ps}, 4\,\mathrm{ps}, 4.9\,\mathrm{ps}$. The vertical dashed cyan line marks $E_\nu = 1525\,\mathrm{eV}$. (a) These data highlight that up to $\approx 3-4$ps there is mostly a drop in diffraction intensity near the peak center, accompanied by a minor shift of the spectral weight to higher photon energies ($I^{2.6\,\mathrm{ps}}_{\mathrm{XRD}}$), indicating lattice compression. (b) After 4 ps the diffraction intensity near the peak center increases and the peak shifts to the lower photon energies ($I^{4.9\,\mathrm{ps}}_{\mathrm{XRD}}$), indicating lattice expansion.

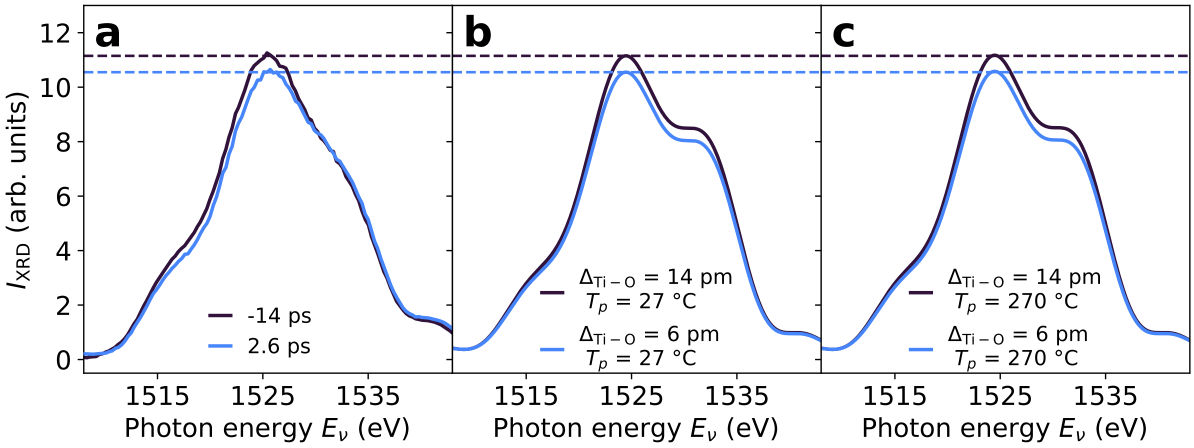

Supplementary Figure 2. **Contribution of Debye-Waller factor.** (a) BTO (001) diffraction peak measured at $t = -14$ ps and $t = 2.6$ ps (same as in Figure 1b). Diffraction intensity simulations that mimic the drop of peak diffraction intensity with $\Delta_{\mathrm{Ti-O}} = 14$ pm and $\Delta_{\mathrm{Ti-O}} = 6$ pm at $T_p = 27\,°\mathrm{C}$ (b) and $T_p = 270\,°\mathrm{C}$ (c). Details of the simulations based on the dynamical theory of diffraction can be found in Ref.[1]. The minimal difference between the diffraction curves in panels (b) and (c) shows that the effect of the average lattice temperature T_p on the diffraction intensity, via the Debye-Waller effect, is negligible in the T_p range relevant to this work ($T_p \leq 270\,°\mathrm{C}$, **Supplementary Figure 3**b). Moreover, the non-monotonic decrease and increase in peak diffraction intensity in the region $0\,\mathrm{ps} < t < 7\,\mathrm{ps}$ is not consistent with the monotonic increase of T_p upon absorption of the pump laser (**Supplementary Figure 3**b).

Supplementary Table 1. Physical constants used in the calculation of two-temperature model and strain model. The film thicknesses d result from θ-2θ measurements (**Supplementary Figure 14**). The penetration depth δ and the reflectivity R_p are discussed in **Supplementary Note 7** B and **Supplementary Note 7** A, respectively. The volumetric heat capacities C_e and C_p of SRO and GSO are calculated from Refs. [2] and [3], respectively, while those of BTO are discussed in **Supplementary Note 1**. The mass density ρ, the bulk modulus B, and the Poisson ratio ν are taken from Ref. [4]. The longitudinal sound velocity is $v = \sqrt{3B(1-\nu)/[\rho(1+\nu)]}$ [5]. The thermal expansion coefficient β of SRO and GSO, and $\beta_{T>T_c}$ of BTO are obtained from data shown in **Supplementary Figure 15**, while $\overline{\beta}_{T<T_c}$ of BTO is a fit parameter, as discussed in **Supplementary Note 2**. The thermal conductivity K_p and diffusivity $D_p = K_p/C_p$ are given in the respective references reported in the Table below. The acoustic reflection coefficient is given by $R_Z = (Z_2 - Z_1)/(Z_2 + Z_1)$, where Z_1 and Z_2 are the acoustic impedances at the two sides of the interface, with $Z = \rho v$ and the strain wave travelling from material 1 to 2. For example, going from BTO to SRO, $R_Z = 0.13$, while going from BTO to the vacuum interface, $R_Z = -1$.

constants	unit	BTO	SRO	GSO
d	nm	34.5	47	0.5×10^6
δ	nm	17.9	21.5	26.1
R_p	-	0.178	0.022	0.018
C_p	$\mathrm{J\,m^{-3}\,K^{-1}}$	2.8×10^6	2.8×10^6 [2]	2.1×10^6 [3]
C_e	$\mathrm{J\,m^{-3}\,K^{-1}}$	4.2×10^4	2.3×10^5 [2]	9×10^4 [3]
ρ [4]	$\mathrm{g\,cm^{-3}}$	5.93	6.46	6.6
m [6]	$\mathrm{g\,mol^{-1}}$	233.19	236.69	250.2
β	$\mathrm{K^{-1}}$	1.33×10^{-5} ($\beta_{T>T_c}$)	1.58×10^{-5}	0.76×10^{-5}
K_p	$\mathrm{W\,m^{-1}\,K^{-1}}$	2.73 [7]	5.72 [2]	2.53 [3]
D_p	$\mathrm{mm^2\,s^{-1}}$	1.11 [7]	2.05 [2]	1.22 [3]
B [4]	GPa	107	166	163
ν [4]	-	0.3	0.31	0.29
v [5]	$\mathrm{m\,s^{-1}}$	5399	6372	6386
Z	$\mathrm{kg\,m^{-2}\,s^{-1}}$	3.2×10^7	4.1×10^7	4.2×10^7
R_Z	-	-1	0.13	0.01

Supplementary Note 1. Two-temperature model

Electron and phonon temperatures, $T_e(z,t)$ and $T_p(z,t)$, result from the analytical solution of the two-temperature model (2TM), consisting of the following coupled equations [8]:

$$\begin{aligned} C_e \frac{\partial T_e}{\partial t} &= -g(T_e - T_p) + S(z,t), \\ C_p \frac{\partial T_p}{\partial t} &= g(T_e - T_p), \end{aligned} \tag{1}$$

where C_e and C_p are volumetric heat capacities, respectively, and g is the electron-phonon coupling. The source term $S(z,t)$ represents the absorbed power density ($\mathrm{W\,m^{-3}}$) of the pump laser in our sample as a function of the pump-probe delay t and the distance z from the BTO surface. It is defined as $S(z,t) = F_{\mathrm{abs}} \exp\left(-z/(\delta \cos\theta_t)\right) \exp\left(-t^2/(2\tau_{\mathrm{OL}}^2)\right)/(\delta\tau_{\mathrm{OL}})$, where F_{abs} is the pump absorbed fluence (**Supplementary Note 7** A), δ is the penetration depth (**Supplementary Note 7** B), θ_t is the angle of the transmitted optical beam with respect to the surface normal, and τ_{OL} is the optical laser pulse duration (**Supplementary Note 10**).

In general, Supplementary Equation (1) contains also the diffusion terms $K_e \partial^2 T_e/\partial z^2$ and $K_p \partial^2 T_p/\partial z^2$, where K_e and K_p are carrier and thermal conductivity, respectively. In the particular case of BTO, carrier and thermal diffusion terms can be neglected because the parameters $D_e/(\nu\delta_{\mathrm{BTO}}) = 2.5\times10^{-5}$ and $D_p/(\nu\delta_{\mathrm{BTO}}) = 1.1\times10^{-2}$ are relatively small. Here, the electron diffusion coefficient is calculated from the Einstein relation $D_e = \mu k_B T/q = 2.6\times10^{-7}\,\mathrm{m^2\,s^{-1}}$, where $\mu = 0.1\,\mathrm{cm^2\,V^{-1}\,s^{-1}}$ is the BTO electron mobility [9], k_B is the Boltzmann constant, T is the sample temperature and q is the electron charge, while the BTO thermal diffusivity [7] is $D_p = 1.11\,\mathrm{mm^2\,s^{-1}}$.

We turn now to discuss the volumetric heat capacities C_e and C_p. Electron and phonon volumetric heat capacities of SRO and GSO are taken from Refs. [2, 3], assuming $T = 300\,\mathrm{K}$. This assumption is justified by the fact that in GSO the transmittance of the 266 nm beam is essentially 0%, while in the SRO thin film only 12% of the incident fluence is absorbed. For the absorbed fluence $F_{\mathrm{abs}} = 0.33\,\mathrm{mJ\,cm^{-2}}$ (**Supplementary Note 7** A), the maximum temperature increase [5] in SRO is $\Delta T = F_{\mathrm{abs}}^{\mathrm{SRO}}/(\delta_{\mathrm{SRO}} C_p^{\mathrm{SRO}}) = 55\,\mathrm{K}$, with a consequent increase of C_p^{SRO} of only 2%. Conversely, C_e^{SRO} increases by 18%, but the absolute value remains one order of magnitude smaller than C_p^{SRO}. The volumetric heat capacity C_p^{BTO} as a function of T is reported in Ref. [10]. Given the weak temperature dependence for $T >$

300 K, we consider $C_p^{\mathrm{BTO}} = 2.8 \times 10^6\,\mathrm{J\,m^{-3}\,K^{-1}}$ and calculate $\Delta T = F_{\mathrm{abs}}^{\mathrm{BTO}}/(\delta_{\mathrm{BTO}} C_p^{\mathrm{BTO}}) =$ 375 K and 196 K, for $F_{\mathrm{abs}}^{\mathrm{BTO}} = 1.88\,\mathrm{mJ\,cm^{-2}}$ and $0.98\,\mathrm{mJ\,cm^{-2}}$ (**Supplementary Note 7** A), respectively. Due to the weak dependence on T, the average of C_p^{BTO} between 300 K and $300\,\mathrm{K} + \Delta T$ yields the self-consistent result of $C_p^{\mathrm{BTO}} = 2.8 \times 10^6\,\mathrm{J\,m^{-3}\,K^{-1}}$ [10]. Finally, to estimate C_e^{BTO}, we rely on the available data from other titanates, e.g. $SrTiO_3$ [11] and $CaTiO_3$ [12], and observe that they have an average ratio $C_e/C_p = 0.015$ for the T ranges considered here. This yields $C_e^{\mathrm{BTO}} = 4.2 \times 10^4\,\mathrm{J\,m^{-3}\,K^{-1}}$. Although C_e^{BTO} is a temperature-dependent quantity, for simplicity, we assume it to be constant here. For completeness, all the physical constants employed in the solution of the 2TM are reported in **Supplementary Table 1**.

We focus now on the analytical solution of the 2TM. The second equation of Supplementary Equation (1) can be written as:

$$T_e = \frac{C_p}{g}\frac{\partial T_p}{\partial t} + T_p, \tag{2}$$

which is then inserted in the first equation of Supplementary Equation (1) and yields the following second order differential equation of T_p:

$$\frac{C_e C_p}{g}\frac{\partial^2 T_p}{\partial t^2} + (C_e + C_p)\frac{\partial T_p}{\partial t} = S(z,t). \tag{3}$$

Given the following initial and boundary conditions: $T_e(z,0) = T_p(z,0) = T_0 = 300\,\mathrm{K}$, $\partial T_p/\partial z(0,t) = 0$, $\partial T_p/\partial t(z,0) = 0$, we can derive the lattice temperature $T_p(z,t)$ as an analytical solution of Supplementary Equation (3):

$$\begin{aligned} T_p(z,t) = T_0 &+ \frac{\sqrt{\pi}\mathcal{C}\tau_{\mathrm{OL}}}{\sqrt{2}\mathcal{B}} \exp\left(\frac{\tau_{\mathrm{OL}}^2\mathcal{B}^2}{2\mathcal{A}^2}\right) \mathrm{erf}\left(\frac{\mathcal{B}\tau_{\mathrm{OL}}}{\sqrt{2}A} - \frac{t}{\sqrt{2}\tau_{\mathrm{OL}}}\right) \exp\left(-\frac{z}{\delta}\right) \exp\left(-\frac{\mathcal{B}}{\mathcal{A}}t\right) \\ &+ \frac{\sqrt{\pi}\mathcal{C}\tau_{\mathrm{OL}}}{\sqrt{2}\mathcal{B}} \exp\left(-\frac{z}{\delta}\right) \mathrm{erf}\left(\frac{1}{\sqrt{2}\tau_{\mathrm{OL}}}t\right) \\ &- \frac{\sqrt{\pi}\mathcal{C}\tau_{\mathrm{OL}}}{\sqrt{2}\mathcal{B}} \exp\left(\frac{\tau_{\mathrm{OL}}^2\mathcal{B}^2}{2\mathcal{A}^2}\right) \mathrm{erf}\left(\frac{\mathcal{B}\tau_{\mathrm{OL}}}{\sqrt{2}A}\right) \exp\left(-\frac{z}{\delta}\right) \exp\left(-\frac{\mathcal{B}}{\mathcal{A}}t\right), \end{aligned} \tag{4}$$

where $\mathcal{A} = C_e C_p/g$, $\mathcal{B} = C_e + C_p$, and $\mathcal{C} = (1/\delta)F_{\mathrm{abs}}$. Substituting Supplementary Equation

(4) in Supplementary Equation (2) we obtain the electron temperature $T_e(z,t)$:

$$\begin{aligned}T_e(z,t) = T_0 - \frac{\sqrt{\pi}\mathcal{C}\tau_{\mathrm{OL}}}{\sqrt{2}\mathcal{B}}\frac{C_p}{C_e}\exp\left(\frac{\tau_{\mathrm{OL}}^2\mathcal{B}^2}{2\mathcal{A}^2}\right)\mathrm{erf}\left(\frac{\mathcal{B}\tau_{\mathrm{OL}}}{\sqrt{2}A} - \frac{t}{\sqrt{2}\tau_{\mathrm{OL}}}\right)\exp\left(-\frac{z}{\delta}\right)\exp\left(-\frac{\mathcal{B}}{\mathcal{A}}t\right)\\ +\frac{\sqrt{\pi}\mathcal{C}\tau_{\mathrm{OL}}}{\sqrt{2}\mathcal{B}}\exp\left(-\frac{z}{\delta}\right)\mathrm{erf}\left(\frac{1}{\sqrt{2}\tau_{\mathrm{OL}}}t\right)\\ +\frac{\sqrt{\pi}\mathcal{C}\tau_{\mathrm{OL}}}{\sqrt{2}\mathcal{B}}\frac{C_p}{C_e}\exp\left(\frac{\tau_{\mathrm{OL}}^2\mathcal{B}^2}{2\mathcal{A}^2}\right)\mathrm{erf}\left(\frac{\mathcal{B}\tau_{\mathrm{OL}}}{\sqrt{2}\mathcal{A}}\right)\exp\left(-\frac{z}{\delta}\right)\exp\left(-\frac{\mathcal{B}}{\mathcal{A}}t\right).\end{aligned} \tag{5}$$

The dependence of T_e and T_p (averaged over d_{BTO}) on the delay t is reported in **Supplementary Figure 3** for the two incident pump fluences $F_{\mathrm{in}} = 2.7\,\mathrm{mJ\,cm^{-2}}$ and $1.4\,\mathrm{mJ\,cm^{-2}}$ and the corresponding fit parameter g (**Supplementary Table 2**), while **Supplementary Figure 7**a and **Supplementary Figure 8**a report the 2D maps of $T_p(z,t)$ as a function of delay t and depth z at the respective fluences.

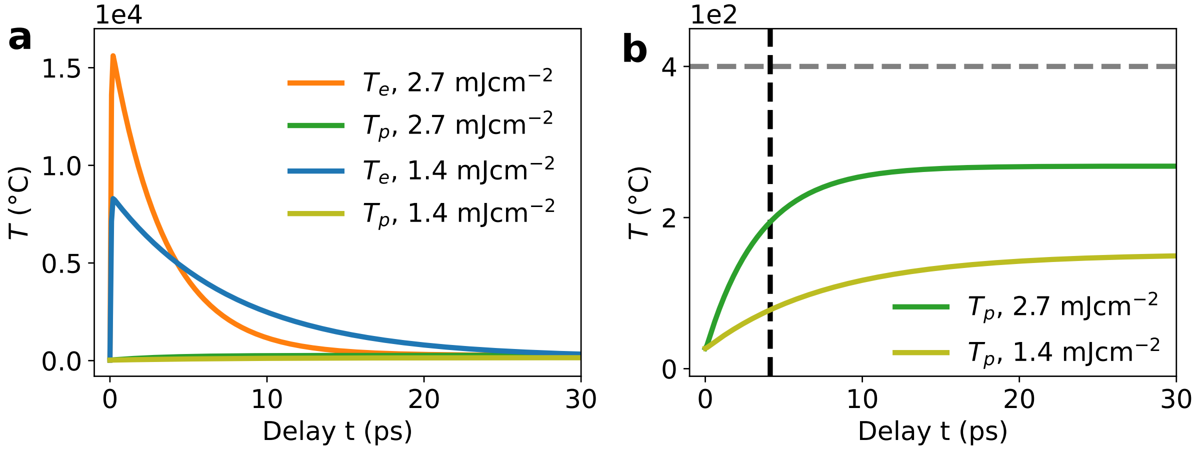

Supplementary Figure 3. Electron and lattice temperature as a function of delay t. (a) T_e and T_p averaged over d_{BTO} as a function of delay t for the incident pump fluences $F_{\mathrm{in}} = 1.4\,\mathrm{mJ\,cm^{-2}}$ and $F_{\mathrm{in}} = 2.7\,\mathrm{mJ\,cm^{-2}}$, calculated as detailed in **Supplementary Note 1**. (b) the same as panel (a) with focus on the temperature range of T_p. The gray dashed line indicates $T_c = 400\,^{\circ}\mathrm{C}$ (**Supplementary Figure 15**).

Supplementary Note 2. Strain model

The total stress experienced by our sample upon optical laser excitation can be written as [13, 14]:

$$\sigma = \rho v^2 \eta + \sigma_{DP}\left(T_e, \frac{\partial E_g}{\partial p}\right) + \sigma_{TE}(T_p, \beta), \tag{6}$$

where $Y = \rho v^2$ is the Young's modulus, ρ is the mass density and v is the longitudinal speed of sound (**Supplementary Table 1**). The first term in Supplementary Equation (6) indicates the direct relationship between the stress σ, a force that induces a deformation of the material, and the strain η that represents the resulting deformation of the material. The stress σ causes the generation of a strain wave that propagates through the material and across the interface to the layer below. In Supplementary Equation (6), $\partial E_g/\partial p$ indicates the variation of the bandgap as a function of the electronic pressure, and β is the thermal expansion coefficient.

Supplementary Equation (6) can be written more explicitly as [13]:

$$\sigma(z,t) = \rho v^2 \eta(z,t) - n_e B \frac{\partial E_g}{\partial p} - 3B\beta(T_p(z,t) - T_0), \tag{7}$$

where $n_e = C_e(T_e(z,t) - T_0)/(E - E_g)$ is the carrier density [14] (with unit m^{-3}), B is the bulk modulus (**Supplementary Table 1**), $T_0 = 300\,\mathrm{K}$ is the sample temperature at equilibrium, and $n_e(E - E_g)$ is the total energy density transferred from the optical photons to the electronic subsystem [14]. We consider here only the dependence of T_e, T_p and η on the z direction (sample depth). This one-dimensional approximation is justified by the large ratio between the laser excited area and the BTO thickness, leading to the film contraction/expansion only along the surface normal on the few tens of picoseconds timescale.

The relation between the stress σ and the atomic displacement u is described by the following one-dimensional lattice strain wave equation [5]:

$$\rho \frac{\partial^2 u(z,t)}{\partial t^2} = \frac{\partial \sigma(z,t)}{\partial z}, \tag{8}$$

which can be recast as a function of $\eta(z,t) = \partial u(z,t)/\partial z$:

$$\frac{\partial^2 \eta(z,t)}{\partial t^2} = \frac{1}{\rho}\frac{\partial^2 \sigma(z,t)}{\partial z^2}. \tag{9}$$

Substituting Supplementary Equation (7) in Supplementary Equation (9) yields:

$$\frac{\partial^2 \eta(z,t)}{\partial t^2} = v^2 \frac{\partial^2 \eta(z,t)}{\partial z^2} - \frac{C_e}{\rho(E - E_g)\delta^2} B \frac{\partial E_g}{\partial p}(T_e(z,t) - T_0) - \frac{3B\beta}{\rho\delta^2}(T_p(z,t) - T_0). \tag{10}$$

Given the initial conditions [5] $\eta(z,0) = 0$, $\partial\eta/\partial t(z,0) = 0$, $\sigma(0,t) = 0$, Supplementary Equation (10) provides the out-of-plane strain profile $\eta(z,t)$ by solving the following analytical integrals [15]:

$$\eta(z,t) = \begin{cases} \frac{1}{2v}\int_0^t \int_{z-v(t-\tau)}^{z+v(t-\tau)} \mathfrak{F}(\xi,\tau)d\xi d\tau, & \text{for } t < \frac{z}{v} \\ \frac{1}{2v}\int_0^{t-\frac{z}{v}} \int_{v(t-\tau)-z}^{z+v(t-\tau)} \mathfrak{F}(\xi,\tau)d\xi d\tau & \\ +\frac{1}{2v}\int_{t-\frac{z}{v}}^{t} \int_{z-v(t-\tau)}^{z+v(t-\tau)} \mathfrak{F}(\xi,\tau)d\xi d\tau + \mathfrak{F}(0,t-z/v)\frac{\delta^2}{v^2}, & \text{for } t > \frac{z}{v}, \end{cases} \tag{11}$$

where $\mathfrak{F}(z,t) = -C_e B\frac{\partial E_g}{\partial p}(T_e(z,t)-T_0)/[\rho(E-E_g)\delta^2] - 3B\beta(T_p(z,t)-T_0)/(\rho\delta^2)$. Electron and phonon temperatures, $T_e(z,t)$ and $T_p(z,t)$, are determined using the 2TM (**Supplementary Note 1**).

Since the optical pump laser is absorbed mostly in the BTO thin film, but partially also in the SRO layer, there will be two strain waves originating at the vacuum/BTO and the BTO/SRO interfaces. These strain waves propagate through the sample and reflect at each interface, with acoustic reflection coefficients R_Z (**Supplementary Table 1**). The almost identical acoustic impedance of SRO and GSO, leads to a $R_Z = 1\%$ at the SRO/GSO interface. Conversely, the strain wave undergoes a 13% reflection at the BTO/SRO interface and 100% reflection at the interface with vacuum, given the acoustic impedance $Z_{\text{vacuum}} = 0$ (**Supplementary Table 1**). The superposition of the generated and reflected strain waves yields a total strain wave $\eta(z,t)$, which is then averaged over the BTO film thickness to obtain $\overline{\eta}(t) = \int_0^{d_{\text{BTO}}} \eta(z,t)dz/d_{\text{BTO}}$. This quantity is used to fit the experimental average strain data obtained from tr-XRD measurements for $F_{\text{in}} = 2.7\,\text{mJ}\,\text{cm}^{-2}$ (Figure 1d of the main text) and for $F_{\text{in}} = 1.4\,\text{mJ}\,\text{cm}^{-2}$ (**Supplementary Figure 4**), with the resulting fit parameters ($\partial E_g/\partial p$, $\overline{\beta}_{T<T_c}$, and g) reported in **Supplementary Table 2**. The parameter $\partial E_g/\partial p$ has been discussed in the main text, we focus here on the discussion of $\overline{\beta}_{T<T_c}$, and g fit results, while the calculations based on our strain model are presented in **Supplementary Note 2**.

The thermal expansion coefficient $\overline{\beta}_{T<T_c}$ is the fit parameter referring to the average β in the range $T_0 < T < T_c$, with Curie temperature $T_c = 400\,°\text{C}$. While for $T > T_c$, the thermal expansion coefficient β is constant and positive ($\beta_{T>T_c} = 1.33 \times 10^{-5}\,\text{K}^{-1}$), it is positive for $T_0 < T < T^*$, and negative for $T^* < T < T_c$ (**Supplementary Figure 15**). In our sample, the temperature below T_c at which β changes sign is $T^* = 200\,°\text{C}$ (**Supplementary Figure 15**). Based on our model, in the metastable state ($t > 20\,\text{ps}$), only $\approx 7\,\text{nm}$ of the BTO film

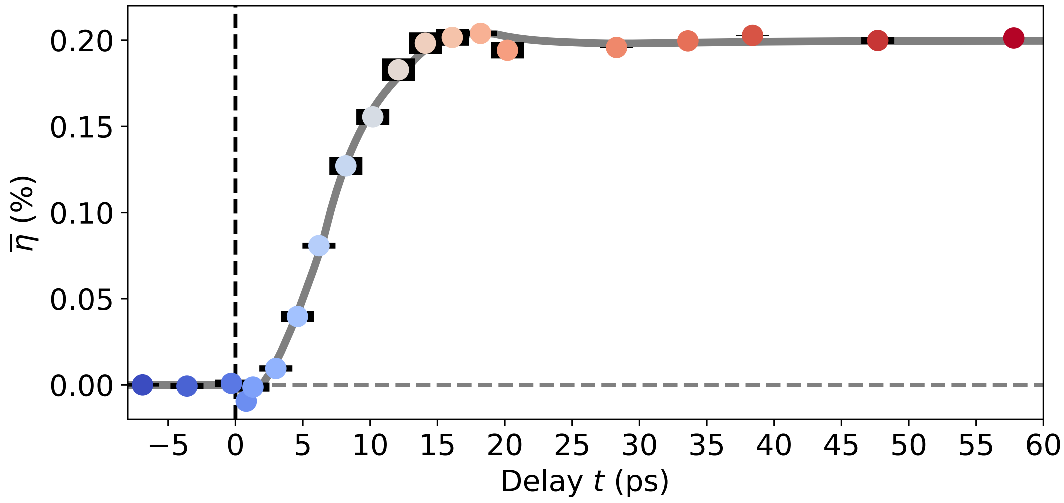

Supplementary Figure 4. $\overline{\eta}(t)$ **with pump fluence** $F_{\mathrm{in}} = 1.4\,\mathrm{mJ\,cm^{-2}}$**.** Average BTO out-of-plane strain $\overline{\eta}(t)$ as a function of pump-probe delay t, measured with the incident pump fluence $F_{\mathrm{in}} = 1.4\,\mathrm{mJ\,cm^{-2}}$. The error bars follow from the determination of c from $I_{\mathrm{XRD}}(E_\nu) \pm \mathrm{SD}$, where SD is the standard deviation. The solid gray line is a fit to the data.

Supplementary Table 2. **Strain model fit results.** Fit results of the experimental $\overline{\eta}(t)$ curves in Figure 1d of the main text and **Supplementary Figure 4**, including $\partial E_g/\partial p$, the thermal expansion coefficient $\overline{\beta}_{T<T_c}$ and the electron-phonon coupling g, measured at two different incident pump fluences F_{in}.

F_{in} ($\mathrm{mJ\,cm^{-2}}$)	$\partial E_g/\partial p$ ($\mathrm{eV\,GPa^{-1}}$)	$\overline{\beta}_{T<T_c}$ ($\mathrm{K^{-1}}$)	g ($\mathrm{W\,m^{-3}\,K^{-1}}$)
1.4	$-1.9(3) \times 10^{-3}$	$8.5(1) \times 10^{-6}$	$6.5(2) \times 10^{15}$
2.7	$-1.9(3) \times 10^{-3}$	$2.5(2) \times 10^{-6}$	$1.2(1) \times 10^{16}$

is at $T^* < T < T_c$ for $F_{\mathrm{in}} = 1.4\,\mathrm{mJ\,cm^{-2}}$, while $\approx 14\,\mathrm{nm}$ of the BTO film is at $T^* < T < T_c$ for $F_{\mathrm{in}} = 2.7\,\mathrm{mJ\,cm^{-2}}$ (**Supplementary Figure 5**). As a result, in the latter case the portion of the sample at $\beta < 0$ is larger than in the former case, thus $\overline{\beta}_{T<T_c}$ is expected to be smaller, as shown by our fit results (**Supplementary Table 2**). At the same time, for both fluences, the larger portion of the sample ($\approx 14 - 27$nm) is at $T_0 < T < T^*$, hence $\overline{\beta}_{T<T_c}$ is expected to be positive, as obtained by our fit results.

The electron-phonon coupling g of the 2TM (**Supplementary Note 1**) is a temperature dependent quantity, however for simplicity in our model it is assumed to be constant,

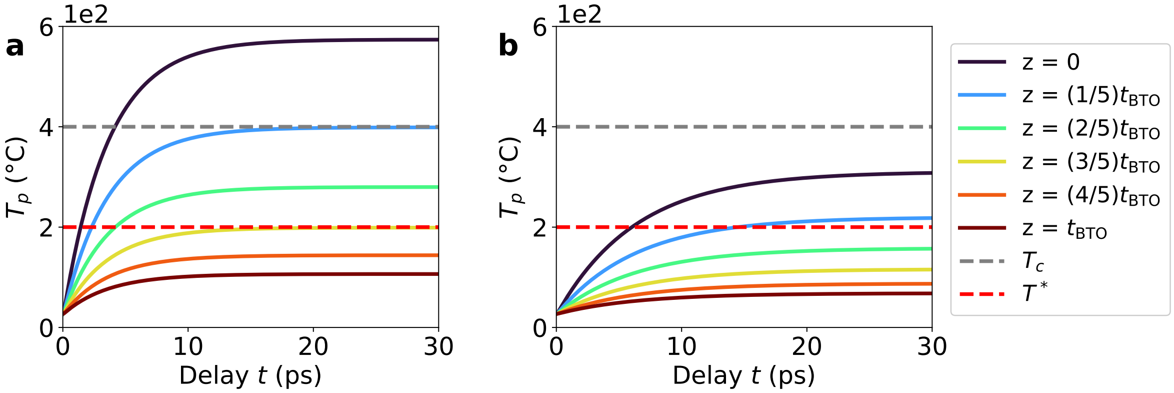

Supplementary Figure 5. **Lattice temperature as a function of delay and depth.** T_p at different depths z from the surface ($z = 0$) as a function of delay t for $F_{\text{in}} = 2.7\,\text{mJ}\,\text{cm}^{-2}$ (a) and $F_{\text{in}} = 1.4\,\text{mJ}\,\text{cm}^{-2}$ (b). The dashed gray[red] line indicates T_c [T^*].

similarly to most current investigations of short-pulse laser excitation [16]. In general, the dependence of g on T_e is related to the electron density of states, and, if the d band electrons are below the Fermi level without crossing it (as in BTO), g increases with T_e. In particular, within the free electron gas model [16], g is linearly dependent on the electron density n_e. This approximation is expected to be valid for relatively low electron temperatures [17], as in our case. In fact, the resulting g fit parameters (**Supplementary Table 2**) scale approximately as the peak electron density given by $n_{e,max} = C_e(T_{e,max} - T_0)/(E - E_g)$ [14] (**Supplementary Figure 12**). Specifically, $n_{e,max} = 1.7 \times 10^{27}\,\text{m}^{-3}$ and $3.2 \times 10^{27}\,\text{m}^{-3}$ for $F_{\text{in}} = 1.4\,\text{mJ}\,\text{cm}^{-2}$ and $2.7\,\text{mJ}\,\text{cm}^{-2}$, respectively. For comparison, the electron density in copper, with one electron per atom in the conduction band ($N_e = 1$), is $n_e^{\text{Cu}} = N_e \rho N_A / M = 8.5 \times 10^{28}\,\text{m}^{-3}$, where $\rho = 8.96\,\text{g}\,\text{cm}^{-3}$ is the mass density [18], $N_A = 6.022 \times 10^{23}$ atoms/mol is the Avogadro's number [19], and $M = 63.55\,\text{g}\,\text{mol}^{-1}$ is the atomic mass [18]. The absolute value of the fit g parameters are of similar order of magnitude as for $SrRuO_3$ [20] and other perovskites [21]. Moreover, it is worth noting that the obtained electron densities and the fit g values are more than one order of magnitude smaller than the corresponding parameters in metals [16, 22, 23], e.g., the electron-phonon coupling in Cu [16] is $g \approx 5 \times 10^{17}\,\text{W}\,\text{m}^{-3}\,\text{K}^{-1}$.

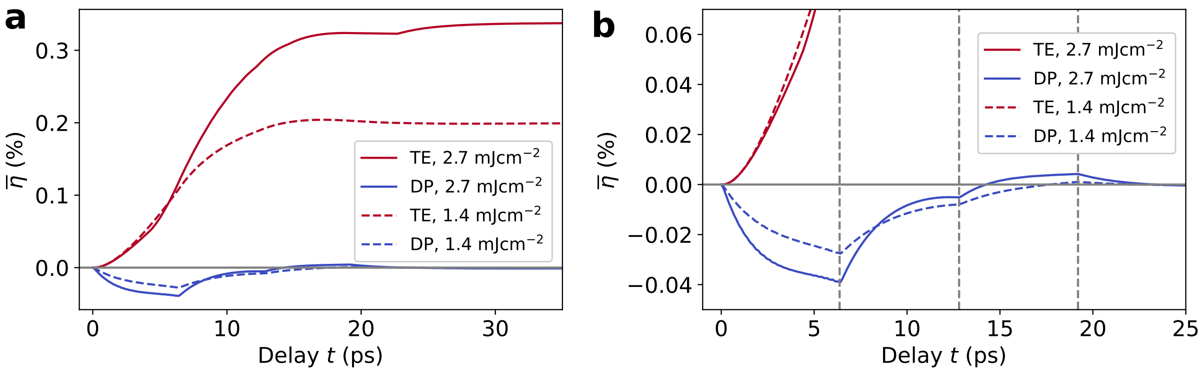

Supplementary Figure 6. **Deformation potential and thermoelastic contributions.** (a) Thermoelastic (TE, red lines) and deformation potential (DP, blue lines) strain contributions at $F_{\text{in}} = 2.7\,\text{mJ}\,\text{cm}^{-2}$ (solid lines) and $F_{\text{in}} = 1.4\,\text{mJ}\,\text{cm}^{-2}$ (dashed lines). (b) same plot as in panel (a) with focus on the strain range $-0.05\% < \overline{\eta}(t) < 0.07\%$ and delay range $-1\,\text{ps} < t < 25\,\text{ps}$. The vertical gray dashed lines at 6.4 ps, 12.8 ps, 19.2 ps mark discontinuities in the DP strain profile, which are due to reflections of the DP strain wave at the BTO/SRO, BTO/air, and again BTO/SRO interfaces (**Supplementary Note 7** A). The time constants result from the speed of sound in BTO (**Supplementary Table 1**) multiplied by the traveled distance, which is d_{BTO}, $2d_{\text{BTO}}$ and $3d_{\text{BTO}}$, respectively. Discontinuities in the TE profile are too small to be seen because of the relatively large absolute value of the average TE strain that is mostly contributed by the BTO region near the surface (**Supplementary Figure 7**b).

Supplementary Note 3. Estimation of the bandgap decrease

To estimate the largest bandgap decrease, first we determine the photoinduced electronic pressure as $\Delta P = \gamma_e C_e \Delta T_e \approx 1.7\,\text{GPa}$ [14], where $\gamma_e \approx 2.6$ [24] is the Grüneisen parameter, C_e is the electronic heat capacity (**Supplementary Table 1**), and $\Delta T_e = 1.56 \times 10^4\,\text{K}$ is the largest increase in electronic temperature of the BTO film averaged over d_{BTO} (**Supplementary Figure 3**). Second, we express the bandgap variation as $\Delta E_g = \frac{\partial E_g}{\partial P} \Delta P \approx -3.2\,\text{meV}$, where $\frac{\partial E_g}{\partial P} = -1.9(3) \times 10^{-3}\,\text{eV}\,\text{GPa}^{-1}$ (**Supplementary Table 2**).

Supplementary Note 4. Temperature, strain and diffraction curve calculations

Given the fit parameters in **Supplementary Table 2**, we calculate the lattice temperature $T_p(z,t)$ in the BTO thin film for $F_{\text{in}} = 2.7\,\text{mJ}\,\text{cm}^{-2}$ (**Supplementary Figure 7**a). Under this condition, 19% of BTO reaches T_c after 20 ps, while for $F_{\text{in}} = 1.4\,\text{mJ}\,\text{cm}^{-2}$ the sample remains always below T_c (**Supplementary Figure 8**a). Hence, a higher pump fluence leads to a larger portion of the BTO reaching higher temperatures and in shorter time. We note that when the pump photon energy E is below or $\lesssim 0.5\,\text{eV}$ above the bandgap E_g, peak power intensities in the range from $\text{kW}\,\text{cm}^{-2}$ to $\text{GW}\,\text{cm}^{-2}$ lead to a sample temperature increase below 50 K [25–29]. In our experiment, given the relatively high peak power intensity in the range 20–39 $\text{GW}\,\text{cm}^{-2}$, and the pump photon energy $\approx 1.2\,\text{eV}$ above the bandgap, sample heating is taken into account.

Supplementary Figure 7b reports the strain map $\eta(z,t)$ in the BTO film based on the results of $T_p(z,t)$ and $T_e(z,t)$, with calculation details reported in **Supplementary Note 2**. Here, it can be clearly seen how the regions of the sample below [above] T_c display smaller [larger] $\eta(z,t)$. In particular, for $t < 3.1\,\text{ps}$ the average strain $\overline{\eta}(t)$ is negative, primarily due to the compressive strain from the deformation potential (Figure 1d from the main text). Moreover, the three straight profiles in the **Supplementary Figure 7**b mark the front of the strain wave propagating in the BTO at the sound speed and then reflected at the interface with SRO and air.

A few selected strain profiles at fixed delays are highlighted in **Supplementary Figure 7**c. Within the first 15 ps after $t = 0\,\text{ps}$ (**Supplementary Note 10**) the strain profile $\eta(z,t)$ undergoes relatively large changes resulting from the varying T_e and T_p, and the corresponding DP and TE contributions (**Supplementary Figure 6**). After $\approx 15\,\text{ps}$ the strain profile reaches a metastable state at least for the following few tens of picoseconds. The maximum average tensile strain of this metastable state is directly proportional to the incident pump fluence and it depends on the film thickness (**Supplementary Figure 7**b-c and **Supplementary Figure 8**b-c).

From $\eta(z,t)$, we calculate $c(z,t) = c_0[\eta(z,t) + 1]$ for each unit cell along z and subsequently the diffraction profiles in the range $0\,\text{ps} < t < 20\,\text{ps}$ (**Supplementary Figure 7**d). A selection of diffraction curves at different t (**Supplementary Figure 7**e) are then compared to the experimental data (**Supplementary Figure 7**f). The main features of

the experimental data, i.e., the shift of the diffraction peak to smaller photon energies, the relative increase/decrease of the diffraction intensity on the low/high photon energy side, the larger broadening and smaller peak diffraction intensity as t increases, are all well reproduced by our simulations. This strongly corroborates the validity of the model employed to describe our data. The initial shift to higher photon energy for $t < 4\,\text{ps}$ is clearly shown by the increase of spectral weight around 1530 eV in **Supplementary Figure 7**d. Conversely, for $t > 4\,\text{ps}$, the average positive strain explains the peak shift to lower photon energy, and the strain gradient is the reason for the change in spectral weight from the high to the low energy side. At the same time, a larger strain gradient leads to broader diffraction curves, and given the conservation of the total area under the curve, to a smaller peak intensity. Qualitatively, the simulated diffraction curves show sharper oscillations, related to the small film thickness, as compared to the experimental ones (**Supplementary Figure 7**e-f). This is the consequence of a slightly larger broadening of the experimental I_{XRD} curves that we assign to an initial strain profile $\eta(z, t = 0\,\text{ps}) \neq 0$, while we assume $\eta(z, t = 0\,\text{ps}) = 0$ throughout the sample for the simulated I_{XRD} curves (**Supplementary Figure 24**). Finally, the discussion above regarding the incident pump fluence $F_{\text{in}} = 2.7\,\text{mJ}\,\text{cm}^{-2}$ applies also to the lower fluence $F_{\text{in}} = 1.4\,\text{mJ}\,\text{cm}^{-2}$. Also in the latter case, we find an excellent agreement between experiments and simulations (**Supplementary Figure 8**e-f).

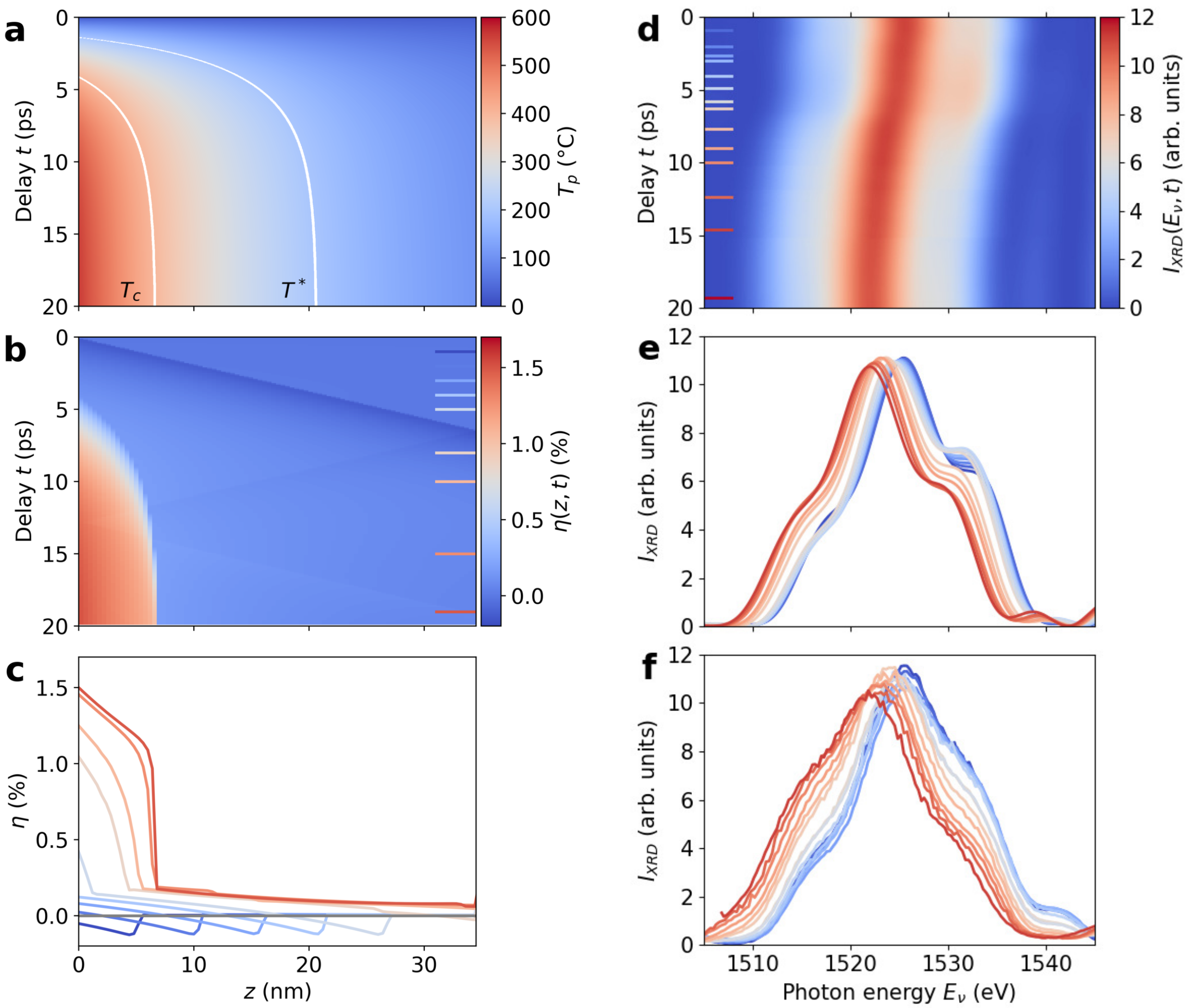

Supplementary Figure 7. Temporal and spatial dependance of lattice temperature and strain, and diffraction curves at fixed delays, with $F_{\text{in}} = 2.7\,\text{mJ cm}^{-2}$. (a) 2D map of the lattice temperature T_p as a function of the delay t and the distance z from the BTO surface. The white lines indicate the Curie temperature $T_c = 400\,°\text{C}$ and $T^* = 200\,°\text{C}$ (**Supplementary Figure 15**). (b) 2D map of the BTO strain $\eta(z,t)$ as a function of t and z. (c) Strain profiles $\eta(z,t)$ at time delays t marked in the panel (b). (d) 2D map of the simulated diffraction curves $I_{\text{XRD}}(t)$ as a function of delay t and photon energy E_ν. (e) Simulated diffraction curves $I_{\text{XRD}}(E_\nu)$ at time delays t marked in panel (d). (f) Experimental diffraction curves at time delays t marked in panel (d). The data shown here refer to the incident pump fluence $F_{\text{in}} = 2.7\,\text{mJ cm}^{-2}$ and result from the solution of the 2TM and the 1D strain wave equation (**Supplementary Note 1** and **Supplementary Note 2**).

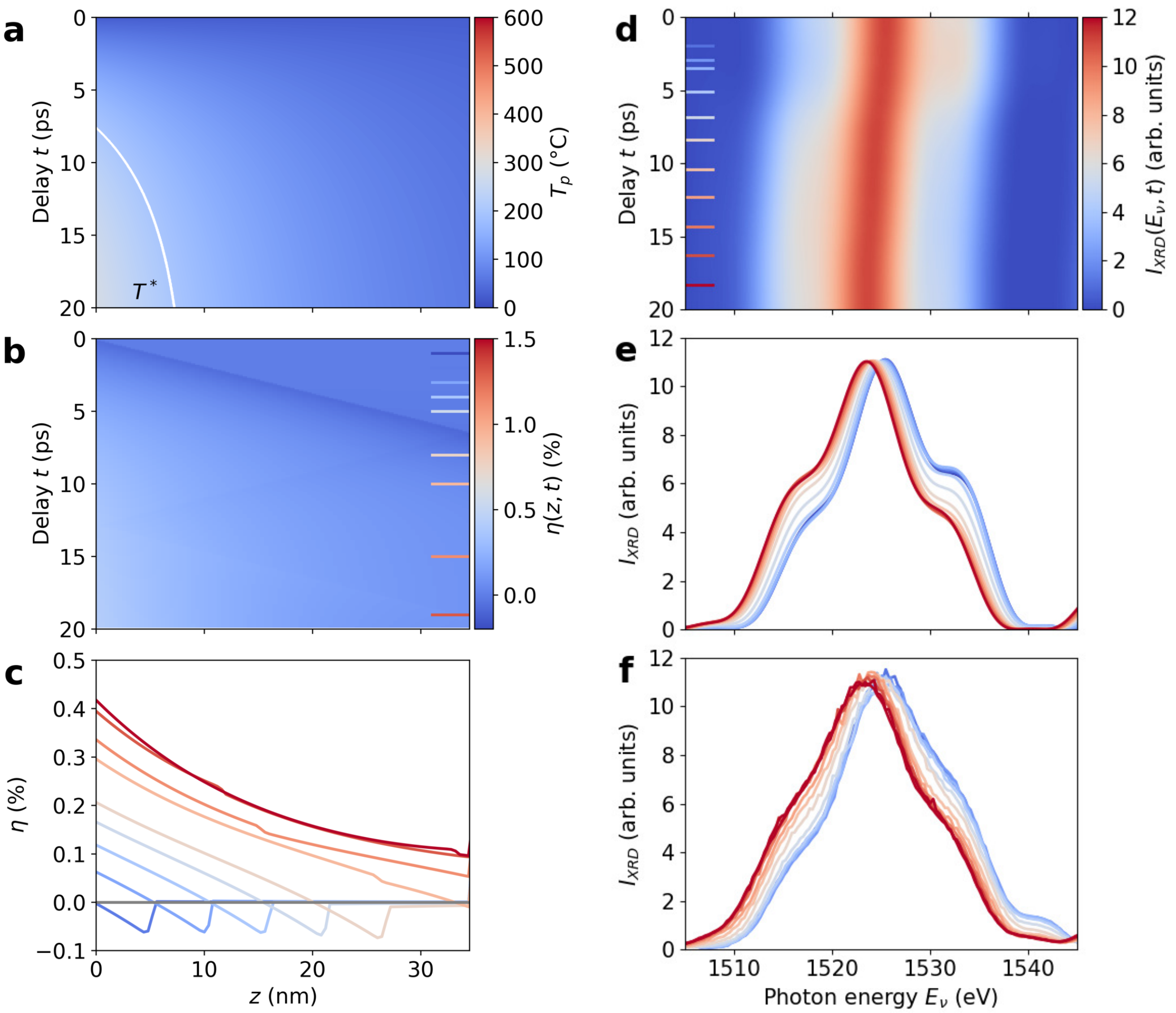

Supplementary Figure 8. Temporal and spatial dependance of lattice temperature and strain, and diffraction curves at fixed delays, with $F_{\mathrm{in}} = 1.4\,\mathrm{mJ\,cm^{-2}}$. (a) 2D map of the lattice temperature T_p as a function of the delay t and the distance z from the BTO surface. The white line indicates the temperature $T^* = 200\,°\mathrm{C}$ (**Supplementary Figure 15**). (b) 2D map of the BTO strain $\eta(z,t)$ as a function of delay t and z. (c) Strain profiles $\eta(z,t)$ at time delays t marked in the panel (b). (d) 2D map of the simulated diffraction curves $I_{\mathrm{XRD}}(t)$ as a function of delay t and photon energy E_ν. (e) Selected diffraction curves $I_{\mathrm{XRD}}(E_\nu)$ at time delays t marked in panel (d). (f) Experimental diffraction curves at time delays t marked in panel (d). The data shown here refer to the incident pump fluence $F_{\mathrm{in}} = 1.4\,\mathrm{mJ\,cm^{-2}}$ and result from the solution of the 2TM and the 1D strain wave equation (**Supplementary Note 1** and **Supplementary Note 2**).

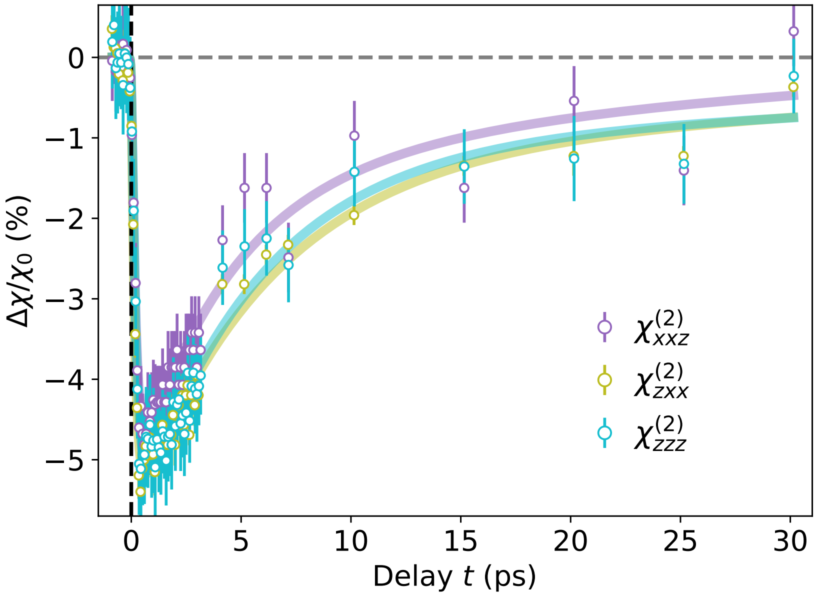

Supplementary Figure 9. Delay dependence of the tensor elements $\chi^{(2)}_{xxz}$, $\chi^{(2)}_{zxx}$, and $\chi^{(2)}_{zzz}$. Relative change $\Delta\chi/\chi_0$ of the tensor elements $\chi^{(2)}_{xxz}$, $\chi^{(2)}_{zxx}$, and $\chi^{(2)}_{zzz}$ as a function of delay t, and corresponding fit curves given by Supplementary Equation (13) (**Supplementary Note 5**). The fit results are: $\tau_0^{\chi_{xxz}} = 200(50)\,\mathrm{fs}$, $\tau_1^{\chi_{xxz}} = 4.2(22)\,\mathrm{ps}$, $\tau_2^{\chi_{xxz}} = 23.5(280)\,\mathrm{ps}$, $\tau_0^{\chi_{zxx}} = 160(30)\,\mathrm{fs}$, $\tau_1^{\chi_{zxx}} = 6.1(24)\,\mathrm{ps}$, $\tau_2^{\chi_{zxx}} = 49(119)\,\mathrm{ps}$, $\tau_0^{\chi_{zzz}} = 220(40)\,\mathrm{fs}$, $\tau_1^{\chi_{zzz}} = 5.6(22)\,\mathrm{ps}$, $\tau_2^{\chi_{zzz}} = 64(219)\,\mathrm{ps}$. The error bars refer to the standard deviation resulting from the fit of the tensor elements. The relatively small difference between $\chi^{(2)}_{zxx}$ (or $\chi^{(2)}_{zzz}$) and $\chi^{(2)}_{xxz}$ is due to the unfavorable experimental geometry with the polarization of the pump laser nearly perpendicular to $\mathbf{P}_s$ (Figure 2a of the main text), and it is expected to increase with the component of the pump polarization along $\mathbf{P}_s$ [30].

Supplementary Note 5. Fit function of tr-XRD, tr-SHG and tr-refl delay scans

The function used to fit the delay scans presented in this work is:

$$F(t) = h(t) * g(t). \tag{12}$$

Supplementary Equation (12) is the convolution of the sum of three exponential decays $h(t) = \sum_{i=1}^{3} A_i \exp\big(-(t-t_0)/\tau_i\big)$, with the Gaussian function $g(t) = \sqrt{2}/(\sigma\sqrt{\pi}) \exp\big(-(t-t_0)^2/(2\sigma^2)\big)$, where $\sigma = \Delta t/(2\sqrt{2\ln 2})$ and Δt is the time resolution (FWHM) of our experiment (**Supplementary Note 10**). The resulting fit function can be written as:

$$F(t) = \sum_{i=1}^{3} A_i \exp\left(\frac{\sigma^2}{2\tau_i^2} - \frac{t-t_0}{\tau_i}\right)\left(1 - \mathrm{erf}\left(\frac{\sigma^2 - \tau_i(t-t_0)}{\sqrt{2}\sigma\tau_i}\right)\right), \tag{13}$$

with the time constants t_0, τ_1, τ_2, and τ_3 and the amplitudes A_1, A_2, and A_3 as fit parameters.

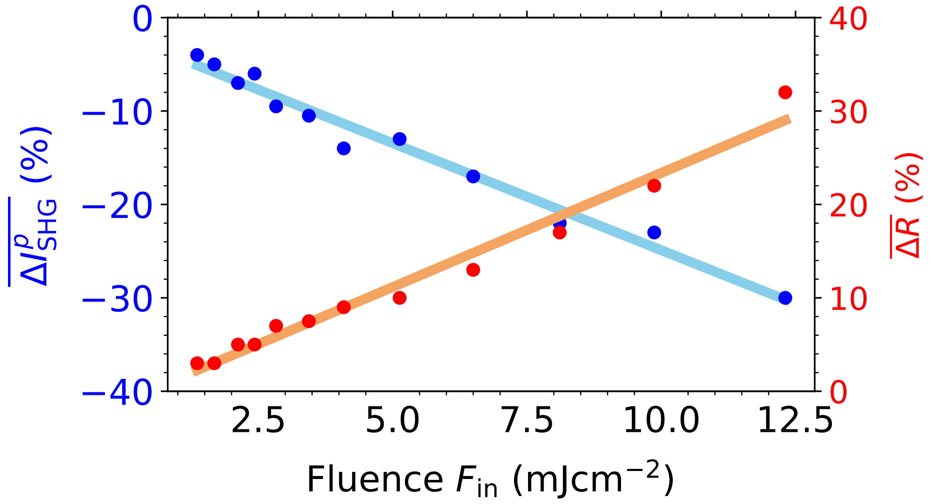

Supplementary Figure 10. **SHG and R maximum relative change.** Maximum relative change of SHG ($\overline{\Delta I^p_{\mathrm{SHG}}}$, blue points) and reflectivity ($\overline{\Delta R}$, red points) as a function of the incident pump fluence F_{in}. The parameters $\overline{\Delta I^p_{\mathrm{SHG}}}$ and $\overline{\Delta R}$ are the average over the delay range $0.32\,\mathrm{ps} < t < 0.73\,\mathrm{ps}$ of $\Delta I^p_{\mathrm{SHG}}/I^p_{\mathrm{SHG}}$ and $\Delta R/R$, respectively (**Supplementary Figure 11**). Blue and red lines are linear fits to the data. The error bars of each data point, equal to the standard deviation of $\Delta I^p_{\mathrm{SHG}}/I^p_{\mathrm{SHG,0}}$ and $\Delta R/R_0$ in the delay range $0.32\,\mathrm{ps} < t < 0.73\,\mathrm{ps}$, are not visible as they are approximately 1%.

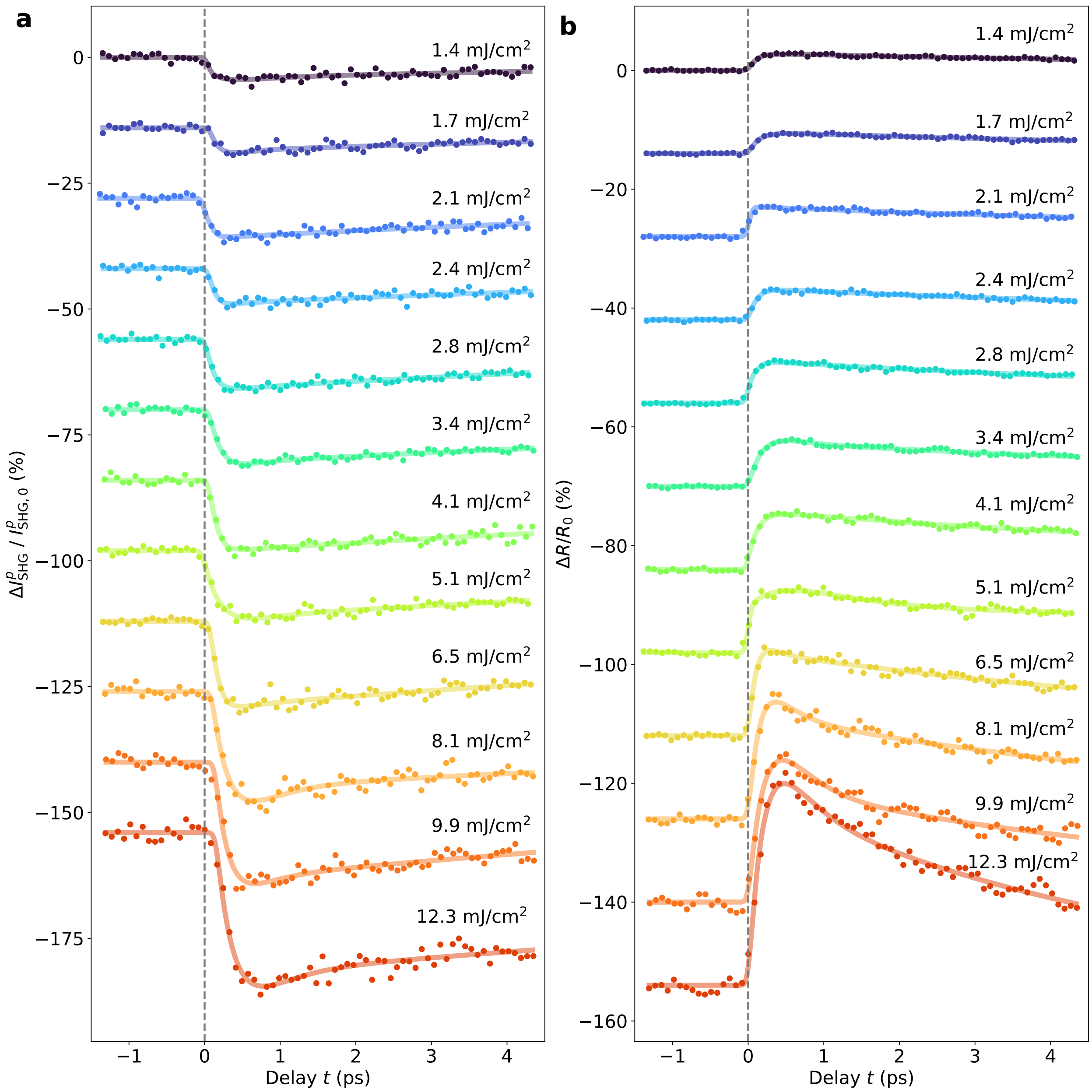

Supplementary Figure 11. **SHG and R time traces at different pump fluences.** $\Delta I^p_{\mathrm{SHG}}/I^p_{\mathrm{SHG},0}$ (a) and $\Delta R/R_0$ (b) as a function of pump-probe delay t, measured at different incident pump laser fluences F_{in} marked on the figure. $\Delta I^p_{\mathrm{SHG}}/I^p_{\mathrm{SHG},0}$ data are measured at $\varphi = 0°$. The maximum relative change of SHG ($\overline{\Delta I^p_{\mathrm{SHG}}}$) and reflectivity ($\overline{\Delta R}$), shown in **Supplementary Figure 10**, are derived from the data reported in this figure. Each time trace has an offset of -14% from the one above.

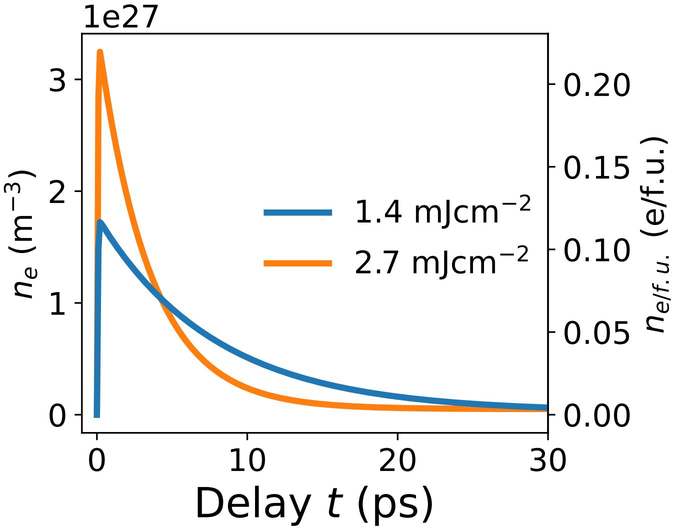

Supplementary Figure 12. **Photoexcited electron density as a function of delay.** Electron density calculated as $n_e = C_e(T_e(t) - T_0)/(E - E_g)$ [14] for two different pump fluences ($1.4\,\mathrm{mJ\,cm^{-2}}$ and $2.7\,\mathrm{mJ\,cm^{-2}}$), and corresponding concentration of photoexcited electrons per formula unit (e/f.u.) $n_{e/f.u.}$, where $n_{e/f.u.} = n_e V$ with the formula unit volume $V = c_0^3 = 67.62 \times 10^{-30}\,\mathrm{m}^3$. Here, $T_e(t)$ refers to the electron temperature averaged over the BTO thickness d_{BTO}.

Supplementary Note 6. Estimation of the carrier diffusion length L_d

The diffusion length L_d, i.e., the distance that a charge carrier can move after generation and before it recombines, is determined by its mobility μ (**Supplementary Note 1**) and lifetime τ as $L_d = \sqrt{k_B T \mu \tau / e}$, where k_B, T and e are the Boltzmann constant, the temperature and the elementary charge, respectively. The electron mobility in BTO[9] is $\mu = 0.1\,\mathrm{cm^2\,V^{-1}\,s^{-1}}$. The lifetime $\tau = 13\,\mathrm{ps}$ is defined as the time required by $\Delta R/R_0$ to drop to $1/e$ of its maximum [31] (see Figure 2f). The temperature range near the BTO/SRO interface goes from 300 K to 417 K (**Supplementary Figure 3**), depending on the pump-probe delay t. The parameters above yield L_d in the range $1.8 - 2.2$ nm, with a maximum of 3 nm near the surface, where T reaches the maximum temperature of 847 K.

Supplementary Note 7. Sample properties

A. Transmittance profile of 266 nm beam in BTO/SRO/GSO

The transmittance of the 266 nm beam in our BTO/SRO/GSO sample, displayed in **Supplementary Figure 13**, is calculated as:

$$\begin{aligned}
\mathcal{T}_{\mathrm{BTO}}(z) &= \exp(-z/(\delta_{\mathrm{BTO}}\cos\theta_t^{\mathrm{BTO}})), \text{for } 0 < z < d_{\mathrm{BTO}}\\
\mathcal{T}_{\mathrm{SRO}}(z) &= \mathcal{T}_{\mathrm{BTO}}(d_{\mathrm{BTO}})(1-R_p^{\mathrm{SRO}})\exp(-z/(\delta_{\mathrm{SRO}}\cos\theta_t^{\mathrm{SRO}})), \text{for } d_{\mathrm{BTO}} < z < d_{\mathrm{BTO}}+d_{\mathrm{SRO}}\\
\mathcal{T}_{\mathrm{GSO}}(z) &= \mathcal{T}_{\mathrm{SRO}}(d_{\mathrm{BTO}}+d_{\mathrm{SRO}})(1-R_p^{\mathrm{GSO}})\exp(-z/(\delta_{\mathrm{GSO}}\cos\theta_t^{\mathrm{GSO}})), \text{for } z > d_{\mathrm{BTO}}+d_{\mathrm{SRO}}.
\end{aligned} \tag{14}$$

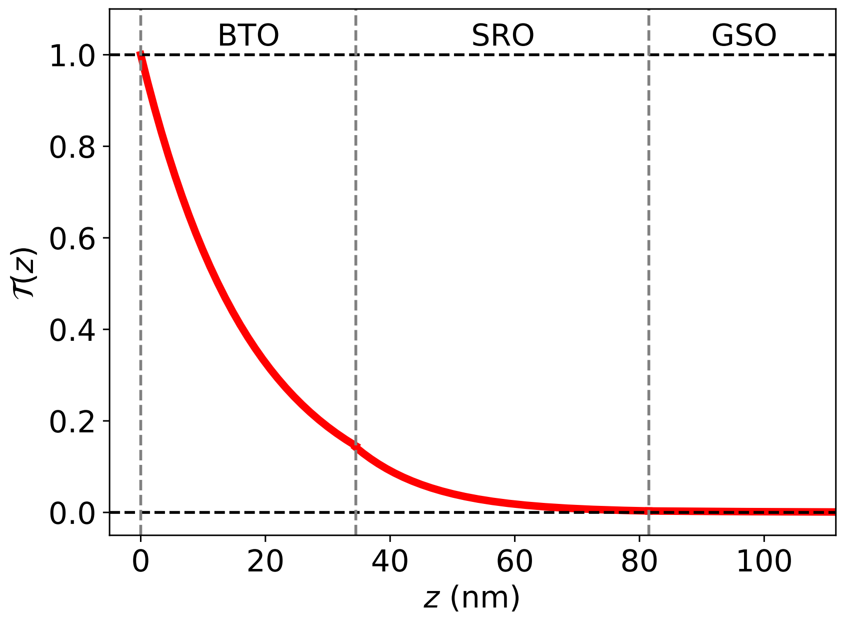

Supplementary Figure 13. Transmittance profile. Transmittance profile $\mathcal{T}(z)$ of the 266 nm pump laser in our BTO/SRO/GSO sample employing pentration depths δ and reflectivities R_p reported in **Supplementary Table 1**. The dashed gray lines mark the BTO surface, the BTO/SRO and the SRO/GSO interfaces.

The penetration depths δ_{BTO}, δ_{SRO} and δ_{GSO} are calculated as detailed in **Supplementary Note 7** B. The reflections at the air-BTO, BTO-SRO, and SRO-GSO interfaces (**Supplementary Table 1**) are determined by using Snell's law $n_1 \sin\theta_i = n_2 \sin\theta_t$ and the Fresnel equation for p-polarized light $R_p = |(n_1\cos\theta_t - n_2\cos\theta_i)/(n_1\cos\theta_t + n_2\cos\theta_i)|^2$.

Here, n_1 and n_2 are the real part of n^* at the two sides of the interface, while θ_i and θ_t are the angles of the incident and the transmitted beam with respect to the surface normal, with $\theta_i^{\mathrm{BTO}} = 4°$. The index of refraction of SRO and GSO are calculated from Ref. [32], as detailed in **Supplementary Note 7** B, while the index of refraction of the BTO thin film is determined from ellipsometry data at 266 nm in Ref. [33].

Given the parameters above (summarized in **Supplementary Table 1**), the absorbed fluences in the BTO and SRO thin films are calculated as:

$$F_{\mathrm{abs}}^{\mathrm{BTO}} = F_{\mathrm{in}}(1 - R_p^{\mathrm{BTO}})(1 - \exp(-d_{\mathrm{BTO}}/(\delta_{\mathrm{BTO}} \cos\theta_t^{\mathrm{BTO}}))) = 0.7F_{\mathrm{in}}, \tag{15}$$

$$F_{\mathrm{abs}}^{\mathrm{SRO}} = F_{\mathrm{in}}(1 - R_p^{\mathrm{BTO}}) \exp[-d_{\mathrm{BTO}}/(\delta_{\mathrm{BTO}} \cos\theta_t^{\mathrm{BTO}})](1 - R_p^{\mathrm{SRO}})[1 - \exp(-d_{\mathrm{SRO}}/(\delta_{\mathrm{SRO}} \cos\theta_t^{\mathrm{SRO}}))] = 0.12F_{\mathrm{in}}, \tag{16}$$

and are reported in **Supplementary Table 3**.

Supplementary Table 3. Absorbed fluences in BTO and SRO thin films. Absorbed fluences in BTO and SRO thin films at two different incident fluences F_{in}.

F_{in} (mJ cm^{-2})	$F_{\mathrm{abs}}^{\mathrm{BTO}}$ (mJ cm^{-2})	$F_{\mathrm{abs}}^{\mathrm{SRO}}$ (mJ cm^{-2})
1.4	0.98	0.17
2.7	1.88	0.33

B. Penetration depths

The absorption coefficient α of the 266 nm pump laser in our BTO thin film is determined based on the study of α as a function of strain in Ref. [33]. For a compressive strain of -0.55%, the penetration depth is $\delta_{\mathrm{BTO}} = 1/\alpha_{\mathrm{BTO}} = 17.9$ nm. The penetration depths in the SRO thin film $\delta_{\mathrm{SRO}} = 21.5$ nm and in the GSO substrate $\delta_{\mathrm{GSO}} = 26.1$ nm are determined from the respective dielectric constants ϵ_1 and ϵ_2, reported in Ref. [32]. Specifically, $\delta = 1/\alpha = \lambda/(4\pi k)$, where $\lambda = 266$ nm is the wavelength of the pump laser, and k is the imaginary part of the complex index of refraction $n^* = n + ik$, with $n = \sqrt{(|\epsilon^*| + \epsilon_1)/2}$, $k = \sqrt{(|\epsilon^*| - \epsilon_1)/2}$, and $|\epsilon^*| = \sqrt{\epsilon_1^2 + \epsilon_2^2}$. The penetration depth of 800 nm and 400 nm

in BTO, $\delta_{\mathrm{BTO}}^{800\,\mathrm{nm}} = 944\,\mathrm{nm}$ and $\delta_{\mathrm{BTO}}^{400\,\mathrm{nm}} = 167\,\mathrm{nm}$, are obtained from the equations above, calculating the complex dielectric function as $\epsilon^* = \epsilon_1 + i\epsilon_2 = \sin\theta_i{}^2[1 + \tan\theta_i{}^2(\frac{1-\rho}{1+\rho})^2]$, where $\rho = \tan\Psi\exp(i\Delta)$, with Ψ and Δ reported in Ref. [33]. Due to the large penetration depths $\delta_{\mathrm{BTO}}^{800\,\mathrm{nm}}$ and $\delta_{\mathrm{BTO}}^{400\,\mathrm{nm}}$, we probe the entire BTO thickness ($d_{\mathrm{BTO}} = 34.5\,\mathrm{nm}$).

Supplementary Note 8. Estimation of the electronic contribution to the ferroelectric polarization magnitude P_s

To quantify the relative contribution of structural ($\Delta P_s^{\eta}/P_s$) and electronic ($\Delta P_s^{n_e}/P_{s,0}$) changes to the total polarization variation out of equilibrium $\Delta P_s/P_{s,0} = (\Delta P_s^{\eta} + \Delta P_s^{n_e})/P_{s,0} = \Delta P_s^{\eta}/P_{s,0}(1 + C_{n_e})$, we first estimate $\Delta P_s^{\eta}/P_{s,0}$, and then derive the relative magnitude of the electronic contribution $C_{n_e} = (\Delta P_s^{n_e}/P_{s,0})/(\Delta P_s^{\eta}/P_{s,0})$. From polarization-electric field hysteresis loops on differently strained BTO thin films grown on a GSO substrate [34], the relative change of the polarization as a function of the strain was determined to be $\Delta P_s^{\eta}/\Delta\eta = 15\,\mu\mathrm{C/cm^2}/\%$. In our data, at short time delays ($t < 4\,\mathrm{ps}$) the strain experiences a marginal change $\Delta\overline{\eta} < 0.02\%$ and ΔP_s is clearly dominated by the presence of photoexcited carriers in the conduction band (Figure 2e-f of the main text). At larger time delays ($t \approx 20\,\mathrm{ps}$), $\overline{\eta}$ reaches the saturation value of 0.34%, which would correspond to an increase in polarization of $\Delta P_s^{\eta} = 5.1\,\mu\mathrm{C\,cm^{-2}}$ ($\Delta P_s^{\eta}/P_{s,0} \approx 25\%$), assuming a polarization at equilibrium of $P_{s,0} \approx 20\,\mu\mathrm{C\,cm^{-2}}$, as found for BTO/SRO/GSO [34]. In contrast, we measure an overall polarization change of $\Delta P_s/P_{s,0} \approx -2.5\%$ (Figure 2e of the main text). As a result, it follows that the electronic contribution C_{n_e}, responsible for the reduced polarization, has $\approx 10\%$ larger magnitude than the structural one even at 20 ps ($C_{n_e} \approx -1.1$).

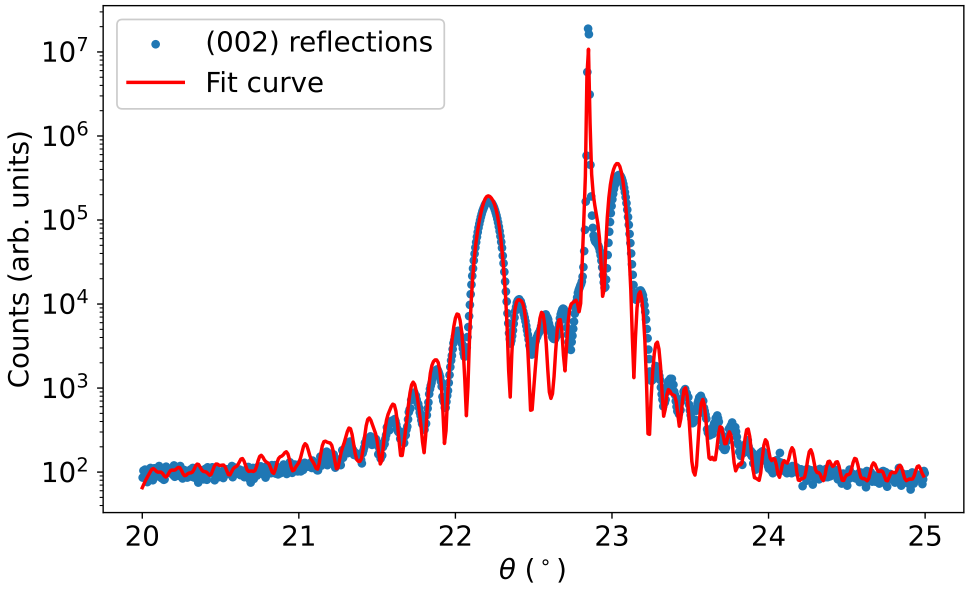

Supplementary Figure 14. **θ-2θ scan.** θ-2θ scan of the as-grown sample around the (002) reflections of BTO and SRO thin films, and the GSO substrate. The measurements are performed using a PANalytical X'Pert Pro diffractometer, and the experimental data are modeled by the dynamical theory of diffraction with BTO and SRO thicknesses as fit parameters, providing $d_{\mathrm{BTO}} = 34.5\,\mathrm{nm}$ and $d_{\mathrm{SRO}} = 47\,\mathrm{nm}$, respectively.

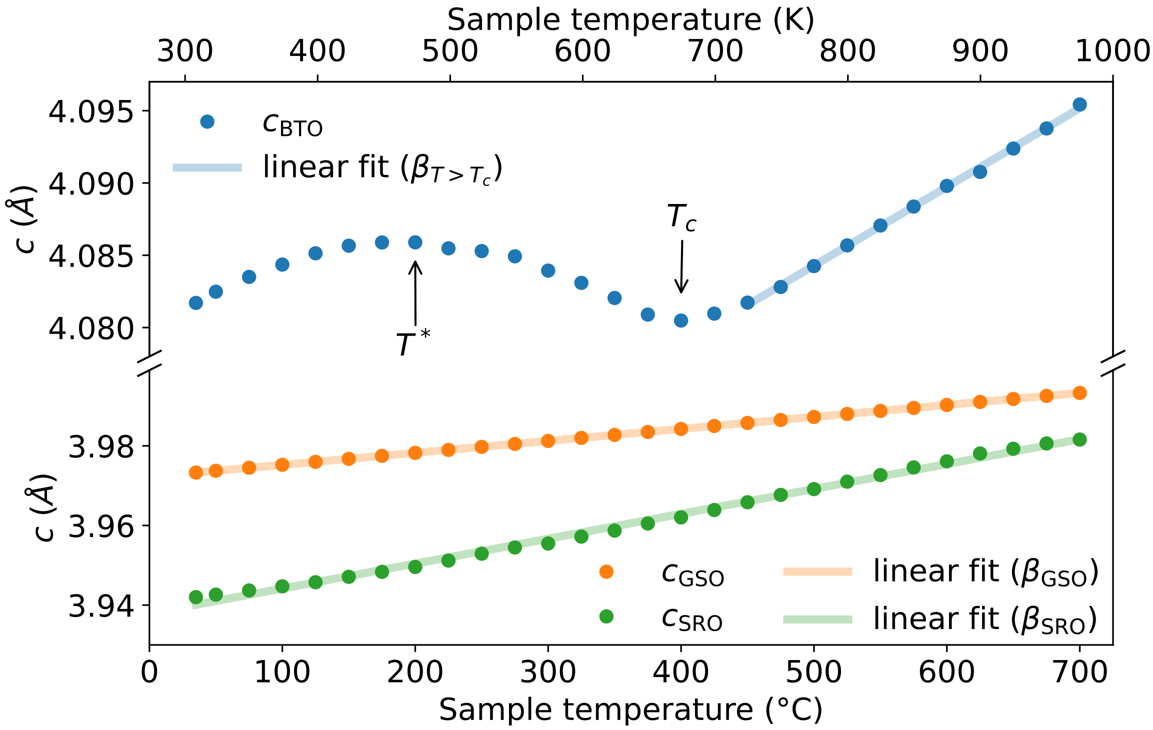

Supplementary Figure 15. c **parameters as a function of sample temperature.** Out-of-plane lattice constant c of BTO (blue points), SRO (green points), and GSO (orange points) as a function of the sample temperature. The c parameters are extracted from θ-2θ scans (similar to **Supplementary Figure 14**) measured at different sample temperatures. The arrows indicate $T^* = 200\,°\mathrm{C}$ and $T_c = 400\,°\mathrm{C}$. The blue line is a linear fit of c_{BTO} data that provides $\beta_{T>T_c} = 1.33 \times 10^{-5}\,\mathrm{K}^{-1}$. The orange and green lines are linear fits of c_{GSO} and c_{SRO} data, which provide $\beta_{\mathrm{GSO}} = 7.6 \times 10^{-6}\,\mathrm{K}^{-1}$ and $\beta_{\mathrm{SRO}} = 1.58 \times 10^{-5}\,\mathrm{K}^{-1}$, respectively.

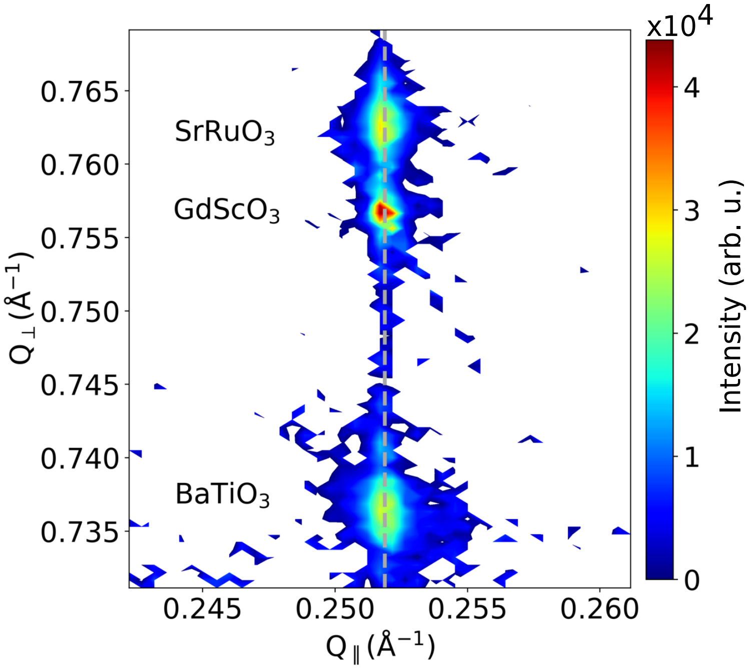

Supplementary Figure 16. **Reciprocal space map.** X-ray reciprocal space map (RSM) of the sample around the ($\bar{1}03$) substrate Bragg peak, measured by a PANalytical X'Pert Pro diffractometer. The vertical gray dashed line indicates the reciprocal lattice parameter $Q_{\|}$ shared by the GSO substrate, BTO, and SRO thin films. The reciprocal lattice parameters $Q_{\|}$ and $Q_{\perp}$ of the intensity peaks are related to the real space in-plane a and out-of-plane c lattice parameters by the following relations [35]: $a = Q_{\|}^{-1} = -\lambda/(2Q_{\mathrm{x}})$ and $c = (Q_{\perp}/3)^{-1} = (3\lambda)/(2Q_{\mathrm{z}})$. Here, $\lambda = 1.5406\,\text{Å}$ is the wavelength of the incident Cu kα radiation, $Q_{\mathrm{x}} = \sin(\theta)\sin(\theta - \omega)$, and $Q_{\mathrm{z}} = \sin(\theta)\cos(\theta - \omega)$, where 2θ is the angle between the incident and the outgoing wavevector, and ω is the angle between the incident wavevector and the sample surface. Fitting the RSM intensity distribution with a pseudo-Voigt function [36] provides $Q_{\|}$ and $Q_{\perp}$ of each diffraction peak, and the following lattice parameters: $a = 3.970\,\text{Å}$, $c_{\mathrm{BTO}} = 4.074\,\text{Å}$, $c_{\mathrm{SRO}} = 3.934\,\text{Å}$, and $c_{\mathrm{GSO}} = 3.964\,\text{Å}$.

Supplementary Note 9. Scanning transmission electron microscopy

High resolution scanning transmission electron microscopy (HR-STEM) was performed on a Cs probe corrected FEI Titan3 G2 60–300 microscope (Thermo Fisher Scientific), equipped with a GIF Quantum ERS from Gatan for electron energy loss spectroscopy (EELS), and a window-less, energy-dispersive X-ray (EDX) system (Super-X) using a four-quadrant silicon drift detector (SDD). The microscope was operated at 300 kV in scanning mode. The electron beam current was set to approximately 50 pA and a convergence semi-angle of 15 mrad was used. The angular range for high-angle annular dark-field (HAADF) imaging was 62.2 to 214.0 mrad. All data for imaging were taken from an area with a relative thickness of $0.6 - 0.7$ in units of inelastic mean free path measured by EELS. Image acquisition and analysis was performed using GMS 3 (version 3.60) by Gatan. EDX spectra were acquired in a thicker area of $0.8 - 1.0$ in units of inelastic mean free path using Velox inherent drift correction, and EDX data were analyzed using Velox by ThermoFisher (version 3.5).

The preparation of the lamella for STEM measurements was done by conventional Focused Ion Beam (FIB) milling on a FEI Nova 200 FIB-SEM, using 10 kV acceleration voltage with a Ga^+ ion beam current of 50 pA in order to minimize sample damage.

Supplementary Figure 17a shows the BTO/SRO interface with single crystalline BTO and no visible stress or dislocations. The BTO/SRO interface is not atomically sharp, but steps do not exceed one unit cell. The SRO/GSO interface is flat and atomically sharp (**Supplementary Figure 17**b). The dark area along the SRO/GSO interface most likely corresponds to differences in surface amorphization arising during the preparation of the lamella for STEM, as elemental analysis did not reveal any compositional variations in this region. At the BTO/SRO interface (**Supplementary Figure 17**c), EDX elemental maps of Ba (**Supplementary Figure 17**d), Ti (**Supplementary Figure 17**e), Sr (**Supplementary Figure 17**f) and Ru (**Supplementary Figure 17**g) confirm that interdiffusion across the interface is below the detection limit.

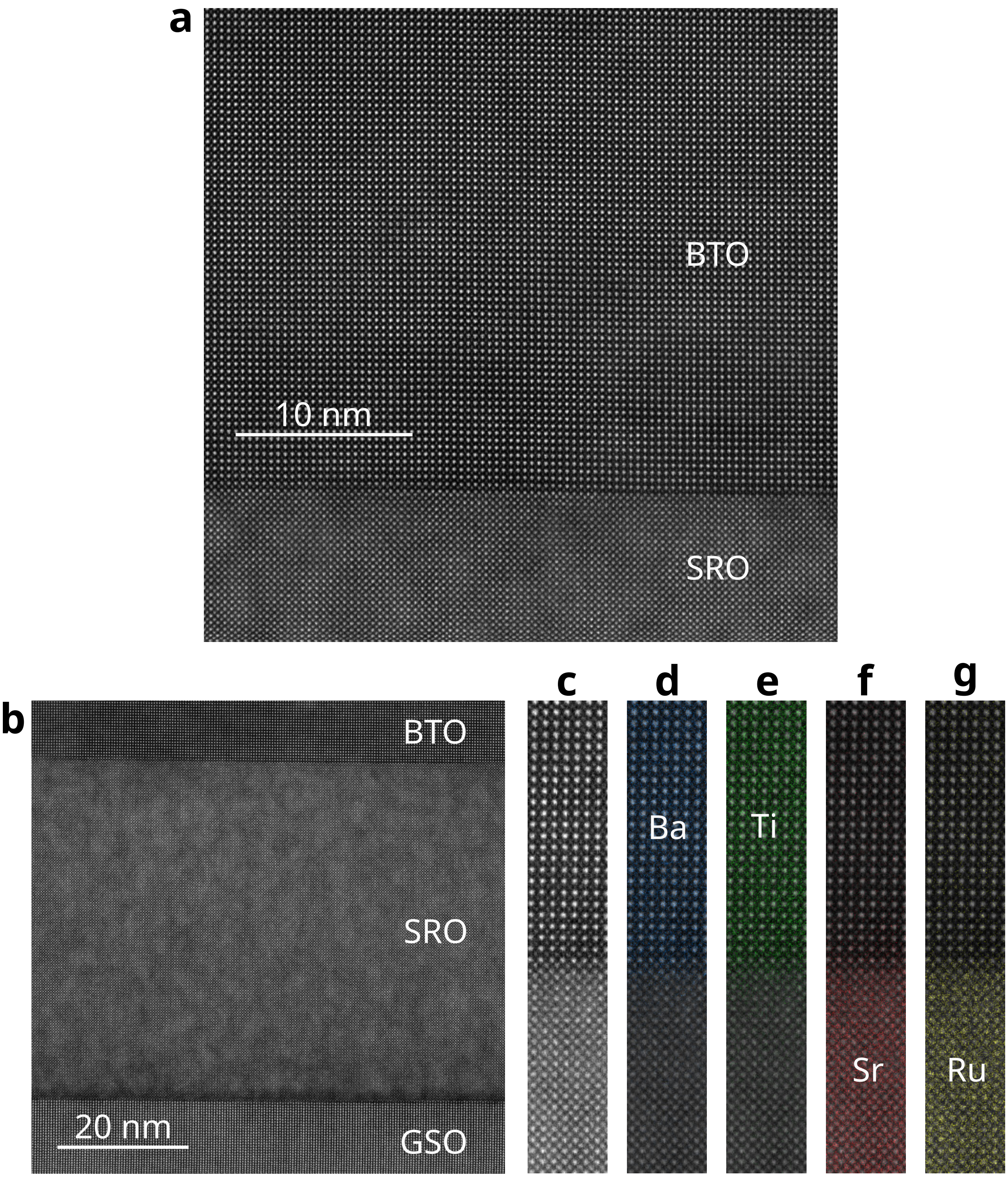

Supplementary Figure 17. **STEM images and EDX elemental maps.** HR-STEM HAADF image of BTO/SRO interface (a), and overview image showing both BTO/SRO and SRO/GSO interfaces (b). HR-STEM HAADF image of the BTO/SRO interface (c) and the corresponding EDX elemental maps of Ba L lines (d), Ti K lines (e), Sr K lines (f), and Ru K lines (g). All acquired images are unfiltered. EDX spectra were acquired with a total pixel dwell time of 12.5 ms.

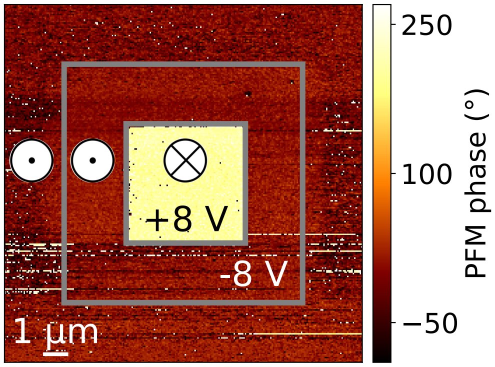

Supplementary Figure 18. **Piezoresponse force microscopy.** Piezoresponse force microscopy (PFM) phase image of the BTO/SRO/GSO sample. Piezoresponse force microscopy in Dual AC Resonance Tracking (DART) mode [37] is used to probe the polarization of as-grown samples and to prove that ferroelectric polarization can be switched by the application of positive or negative voltage between the PFM tip and the SRO electrode. The application of tip voltage 8 V [−8 V], to the regions marked by the gray boxes, forces a downward [upward] polarization $\mathbf{P}_s$ pointing away from the surface (⊗) [toward the surface (⊙)], with the corresponding PFM phase 180° [0°]. The PFM phase in the region where no voltage is applied (beyond the outer gray box) reveals that our as-grown sample has upward polarization $\mathbf{P}_s$ with no indication of multiple domains.

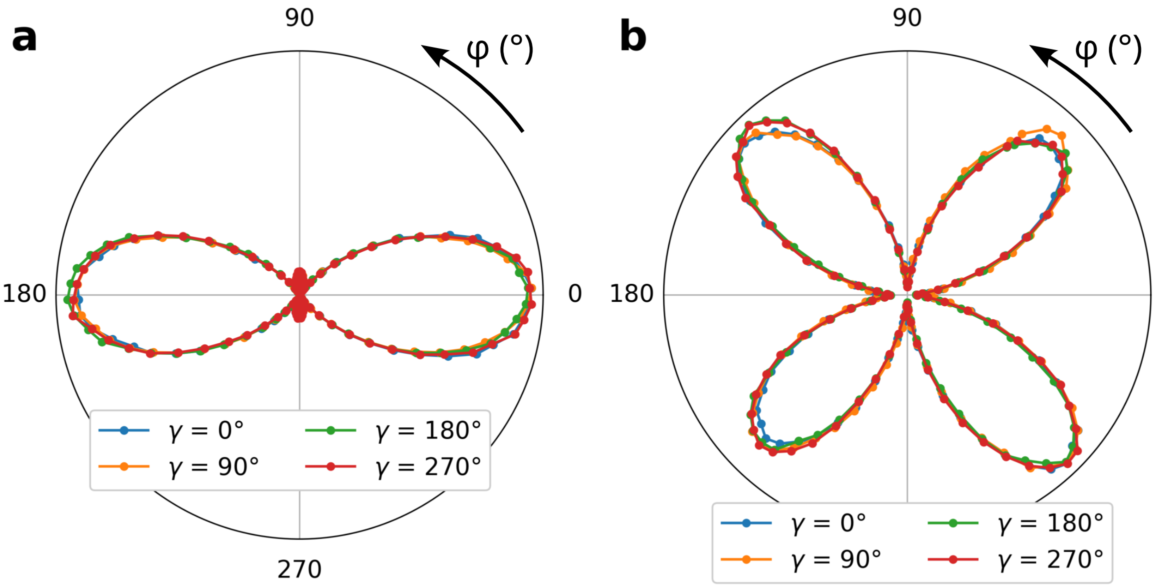

Supplementary Figure 19. **SHG plots at different azimuthal angles.** Polar plots in p-out (a) and s-out (b) configuration measured at the four azimuthal angles $\gamma = 0°, 90°, 180°, 270°$. Regardless of the angle γ, the polar plots measured in the same polarization configuration overlap. This confirms the out-of-plane nature of the spontaneous polarization of our BTO sample. The presence of an in-plane component would manifest as different φ-dependence for different angles γ [38].

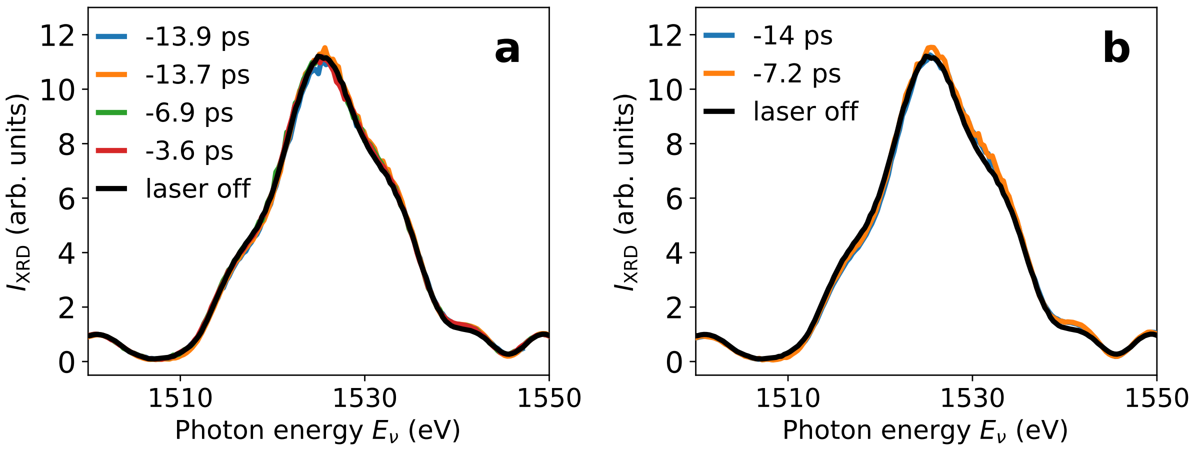

Supplementary Figure 20. Comparison of $I_{\mathrm{XRD}}(E_\nu)$ at negative delay and laser off. Comparison of laser off X-ray diffraction data $I_{\mathrm{XRD}}(E_\nu)$ with data measured at negative delays, at incident fluence $F_{\mathrm{in}} = 1.4\,\mathrm{mJ\,cm^{-2}}$ (a) and $F_{\mathrm{in}} = 2.7\,\mathrm{mJ\,cm^{-2}}$ (b). The difference among different diffraction curves is negligible.

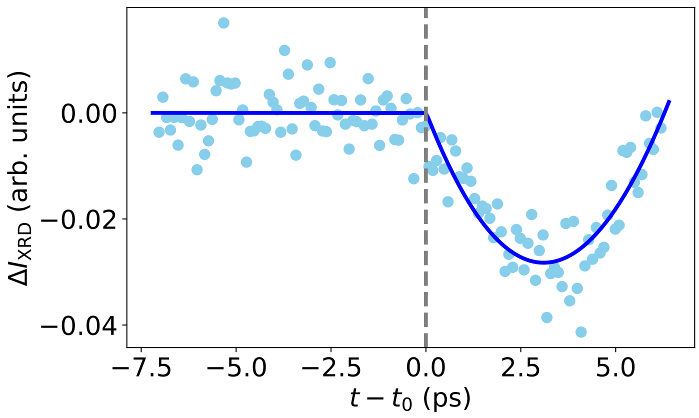

Supplementary Figure 21. **Determination of t_0 in tr-XRD experiments.** The time delay t_0 corresponding to the temporal overlap of XFEL and OL during the tr-XRD measurements was determined by fitting high statistics $\Delta I_{\mathrm{XRD}}(t - t_0)$ data at $E_\nu = 1525\,\mathrm{eV}$ (shown here) using the fit function (13) (**Supplementary Note 5**).

Supplementary Note 10. Bunch arrival time monitor and time resolution of tr-XRD experiments

The bunch arrival time monitor (BAM) at the European XFEL tracks the arrival time of electron bunches in each pulse train, providing invaluable information to improve the time resolution of pump-probe experiments [39, 40]. The BAM measures the electron bunch arrival time with respect to the master clock that is also used to synchronize the pump laser. We employ the most downstream BAM, located at the end of the accelerator tunnel (1932 m from the laser gun) and about 1.5 km upstream of the interaction point at the sample position. In previous time-resolved experiments at the SCS Instrument, it has been observed that the time drifts measured by the BAM are amplified by $17\% - 45\%$ at the interaction point [41]. In practice, this translates in a further correction of up to few tens of femtoseconds. In our experiments, the exact amplification coefficient is not known, thus we assume a direct proportionality between BAM and t_0 at the experiment, where t_0 indicates the temporal overlap between XFEL and optical laser. This assumption is justified by the dynamics of tr-XRD experiments taking place on the picosecond timescale (Figure 1 of the main text).

We measure a standard deviation of the BAM within a pulse train of 10 fs, and a standard deviation of the BAM from train to train of 84 fs. Most importantly, slow drifts of the arrival time of FEL pulses over the course of hours are present and shown in **Supplementary Figure 22** for each recorded run. This indicates that without BAM correction, the time resolution of our experiments might be limited up to ≈ 600 fs, if we average non-consecutive runs. To improve our time resolution, the time delays of our data, indicating the relative time between FEL and optical laser pulses, are corrected for each pulse train according to the average BAM value of the respective pulse train. Thus, the resulting expected time resolution is $\Delta t = \sqrt{\tau_{\mathrm{FEL}}^2 + \sigma_{\tau_{\mathrm{FEL}}}^2 + \tau_{\mathrm{OL}}^2 + \sigma_{\tau_{\mathrm{OL}}}^2} \approx 90$ fs. This assumes an FEL pulse duration of $\tau_{\mathrm{FEL}} \approx 35$ fs, including the nominal 25 fs pulse duration [42] and the pulse stretching at the monochromator of ≈ 10 fs (FWHM) [43], a pulse-to-pulse jitter in the train of $\sigma_{\tau_{\mathrm{FEL}}} \approx 20$ fs, an optical laser pulse duration of $\tau_{\mathrm{OL}} \approx 70$ fs and temporal jitter of $\sigma_{\tau_{\mathrm{OL}}} \approx 30$ fs.

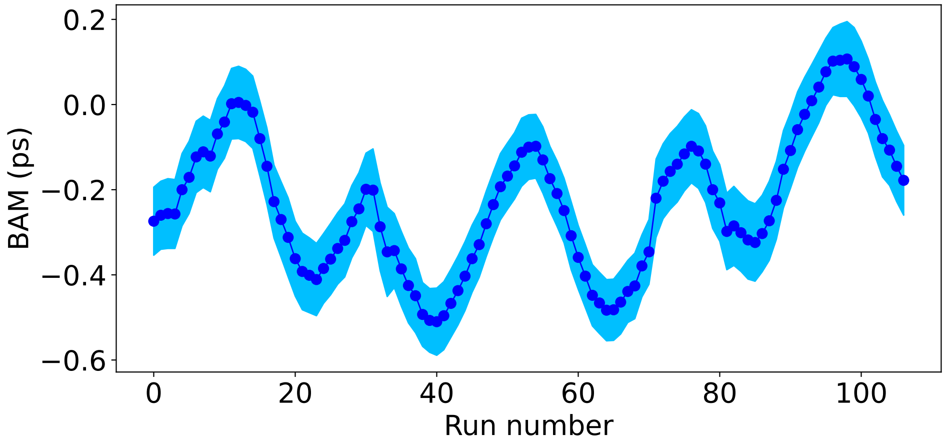

Supplementary Figure 22. **Beam arrival monitor.** Mean (points) and standard deviation (shaded area) of the BAM values of consecutive runs (not evenly spaced in time) recorded within 60 h.

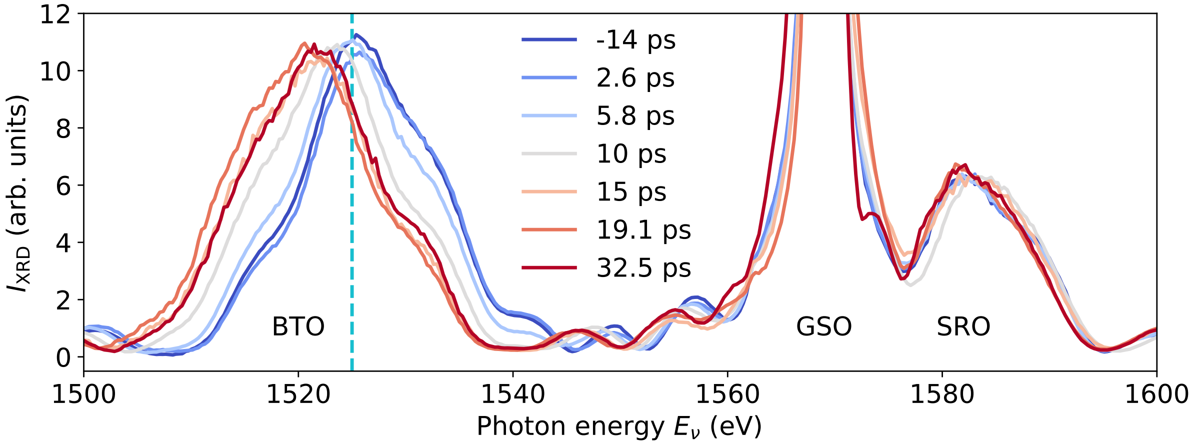

Supplementary Figure 23. **BTO, SRO and GSO (001) diffraction peaks.** Time-resolved (001) Bragg peaks of the BTO/SRO/GSO sample at different time delays t. In contrast to the relatively large energy shifts of the BTO Bragg peak, the SRO and GSO (001) Bragg peaks show much weaker energy shifts, corresponding to changes of the average strain smaller than 0.05% and 0.02%, respectively.

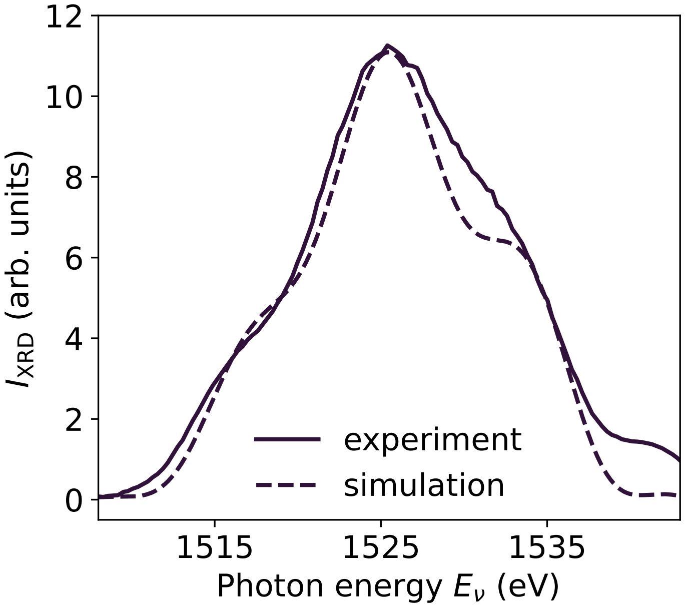

Supplementary Figure 24. Comparison of simulated and experimental $I_{\mathrm{XRD}}(E_\nu)$ at negative delay. Comparison of simulated (dashed line) and experimental (solid line) diffraction curve before the arrival of the pump laser. The broadening of the diffraction curves is calculated as $c_{\mathrm{std}} = (1\,240\,000\,\mathrm{eV\,pm})/(2E_{\nu,\mathrm{std}}\sin\theta)$, where $E_{\nu,\mathrm{std}}$ is the standard deviation of energy values, around the (001) BTO peak, weighted by $I_{\mathrm{XRD}}(E_\nu)$. Here, $c_{\mathrm{std}} = 1.63\,\mathrm{pm}$ and $1.80\,\mathrm{pm}$ for simulated and experimental curves, respectively. This is the consequence of a slightly larger broadening of the experimental I_{XRD} curve due to the presence of an initial strain profile $\overline{\eta} \neq 0$, while we assume $\overline{\eta} = 0$ for the simulated I_{XRD} curve.

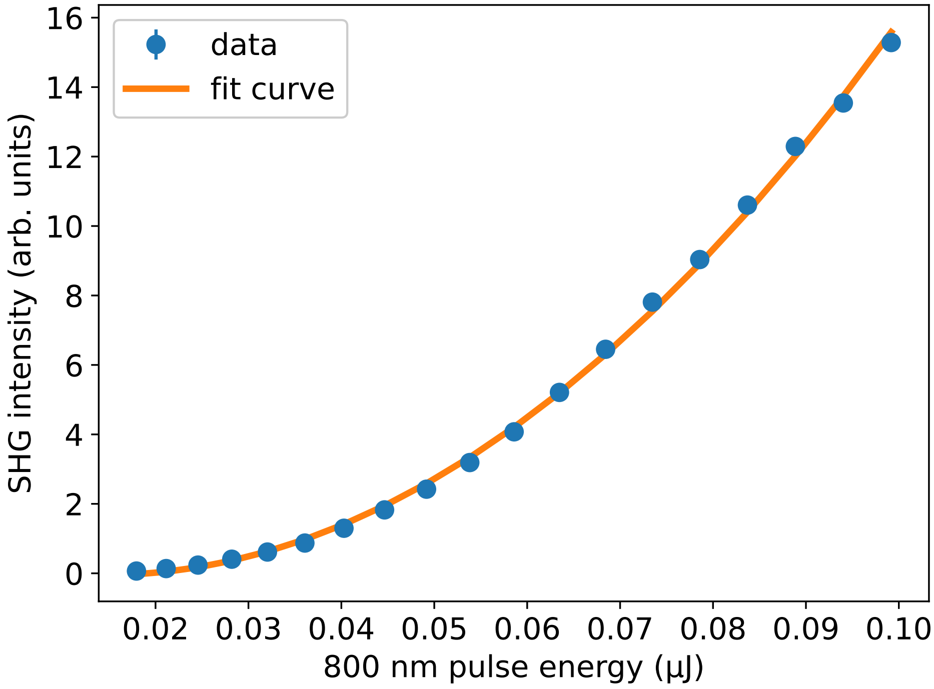

Supplementary Figure 25. **SHG intensity.** SHG intensity measured as a function of the 800 nm probe pulse energy. The fit curve to the data is a polynomial of second order, thus it confirms the nonlinear nature of the signal measured by the photomultiplier.

[1] L. P. Hoang, I. Spasojevic, T.-L. Lee, D. Pesquera, K. Rossnagel, J. Zegenhagen, G. Catalan, I. A. Vartanyants, A. Scherz, and G. Mercurio, Surface polarization profile of ferroelectric thin films probed by X-ray standing waves and photoelectron spectroscopy, Sci. Rep. **14**, 24250 (2024).

[2] S. Yamanaka, T. Maekawa, H. Muta, T. Matsuda, S.-i. Kobayashi, and K. Kurosaki, Thermophysical properties of $SrHfO_3$ and $SrRuO_3$, J. Solid State Chem. **177**, 3484 (2004).

[3] J. Hidde, C. Guguschev, S. Ganschow, and D. Klimm, Thermal conductivity of rare-earth scandates in comparison to other oxidic substrate crystals, J. Alloys Compd. **738**, 415 (2018).

[4] M. de Jong, W. Chen, T. Angsten, A. Jain, R. Notestine, A. Gamst, M. Sluiter, C. Krishna Ande, S. van der Zwaag, J. J. Plata, C. Toher, S. Curtarolo, G. Ceder, K. A. Persson, and M. Asta, Charting the complete elastic properties of inorganic crystalline compounds, Sci. Data **2**, 150009 (2015).

[5] C. Thomsen, H. T. Grahn, H. J. Maris, and J. Tauc, Surface generation and detection of phonons by picosecond light pulses, Phys. Rev. B **34**, 4129 (1986).

[6] I. Mills, International Union of Pure and Applied Chemistry, and International Union of Pure and Applied Chemistry, eds., *Quantities, units, and symbols in physical chemistry*, 2nd ed. (1993).

[7] Y. He, Heat capacity, thermal conductivity, and thermal expansion of barium titanate-based ceramics, Thermochim. Acta **419**, 135 (2004).

[8] S. I. Anisimov, B. L. Kapeliovich, and T. L. Perelman, Electron emission from metal surfaces exposed to ultrashort laser pulses, Zh. Eksp. Teor. Fiz **66**, 375 (1974).

[9] A. Müller and K. H. Härdtl, Ambipolar diffusion phenomena in $BaTiO_3$ and $SrTiO_3$, Appl. Phys. A **49**, 75 (1989).

[10] Z. Wang, M. Yang, and H. Zhang, Strain engineering on electrocaloric effect in $PbTiO_3$ and $BaTiO_3$, Adv. Compos. Hybrid Mater. **4**, 1239 (2021).

[11] D. de Ligny and P. Richet, High-temperature heat capacity and thermal expansion of $SrTiO_3$ and $SrZrO_3$ perovskites, Phys. Rev. B **53**, 3013 (1996).

[12] F. Guyot, P. Richet, P. Courtial, and P. Gillet, High-temperature heat capacity and phase transitions of $CaTiO_3$ perovskite, Phys. Chem. Miner. **20** (1993).

[13] O. B. Wright and V. E. Gusev, Acoustic generation in crystalline silicon with femtosecond optical pulses, Appl. Phys. Lett. **66**, 1190 (1995).

[14] P. Ruello and V. E. Gusev, Physical mechanisms of coherent acoustic phonons generation by ultrafast laser action, Ultrasonics **56**, 21 (2015).

[15] V. S. Vladimirov, ed., *A collection of problems on the equations of mathematical physics* (Springer Berlin Heidelberg, Berlin, Heidelberg, 1986).

[16] Z. Lin, L. V. Zhigilei, and V. Celli, Electron-phonon coupling and electron heat capacity of metals under conditions of strong electron-phonon nonequilibrium, Phys. Rev. B **77**, 075133 (2008).

[17] X. Y. Wang, D. M. Riffe, Y.-S. Lee, and M. C. Downer, Time-resolved electron-temperature measurement in a highly excited gold target using femtosecond thermionic emission, Phys. Rev. B **50**, 8016 (1994).

[18] J. R. Rumble, ed., *CRC Handbook of Chemistry and Physics*, 104th ed. (CRC Press, 2023).

[19] Fundamental Physical Constants from NIST.

[20] K. Wang, B. Zhang, W. Xie, S. Liu, X. Wei, Z. Cai, M. Gu, Y. Tao, T. Yang, C. Zhang, H. Cai, F. Zhang, and X. Wu, Coupling among carriers and phonons in femtosecond laser pulses excited $SrRuO_3$: a promising candidate for optomechanical and optoelectronic applications, ACS Appl. Nano Mater. **2**, 3882 (2019).

[21] C. C. S. Chan, K. Fan, H. Wang, Z. Huang, D. Novko, K. Yan, J. Xu, W. C. H. Choy, I. Lončarić, and K. S. Wong, Uncovering the electron-phonon interplay and dynamical energy-dissipation mechanisms of hot carriers in hybrid lead halide perovskites, Adv. Energy Mater. **11**, 2003071 (2021).

[22] H. E. Elsayed-Ali, T. B. Norris, M. A. Pessot, and G. A. Mourou, Time-resolved observation of electron-phonon relaxation in copper, Phys. Rev. Lett. **58**, 1212 (1987).

[23] J. Hohlfeld, S. S. Wellershoff, J. Güdde, U. Conrad, V. Jähnke, and E. Matthias, Electron and lattice dynamics following optical excitation of metals, Chem. Phys. **251**, 237 (2000).

[24] R. Choithrani, Structural, elastic and thermal properties of $BaTiO_3$, Invertis Journal of Science & Technology **7**, 72 (2014).

[25] D. Daranciang, M. J. Highland, H. Wen, S. M. Young, N. C. Brandt, H. Y. Hwang, M. Vattilana, M. Nicoul, F. Quirin, J. Goodfellow, T. Qi, I. Grinberg, D. M. Fritz, M. Cammarata, D. Zhu, H. T. Lemke, D. A. Walko, E. M. Dufresne, Y. Li, J. Larsson, D. A. Reis, K. Sokolowski-Tinten, K. A. Nelson, A. M. Rappe, P. H. Fuoss, G. B. Stephenson, and A. M. Lindenberg, Ultrafast photovoltaic response in ferroelectric nanolayers, Phys. Rev. Lett. **108**,

087601 (2012).

[26] D. Schick, M. Herzog, H. Wen, P. Chen, C. Adamo, P. Gaal, D. G. Schlom, P. G. Evans, Y. Li, and M. Bargheer, Localized excited charge carriers generate ultrafast inhomogeneous strain in the multiferroic $BiFeO_3$, Phys. Rev. Lett. **112**, 097602 (2014).

[27] S. Matzen, L. Guillemot, T. Maroutian, S. K. K. Patel, H. Wen, A. D. DiChiara, G. Agnus, O. G. Shpyrko, E. E. Fullerton, D. Ravelosona, P. Lecoeur, and R. Kukreja, Tuning ultrafast photoinduced strain in ferroelectric-based devices, Adv. Electron. Mater. **5**, 1800709 (2019).

[28] Y. Ahn, A. S. Everhardt, H. J. Lee, J. Park, A. Pateras, S. Damerio, T. Zhou, A. D. DiChiara, H. Wen, B. Noheda, and P. G. Evans, Dynamic tilting of ferroelectric domain walls caused by optically induced electronic screening, Phys. Rev. Lett. **127**, 097402 (2021).

[29] S. Ganguly, D. Pesquera, D. M. Garcia, U. Saeed, N. Mirzamohammadi, J. Santiso, J. Padilla, J. M. C. Roque, C. Laulhé, F. Berenguer, L. G. Villanueva, and G. Catalan, Photostrictive actuators based on freestanding ferroelectric membranes, Adv. Mater. **36**, 2310198 (2024).

[30] J. Chen, L. Hong, B. Huang, and H. Xiang, Ferroelectric switching assisted by laser illumination, Phys. Rev. B **109**, 094102 (2024).

[31] Z. Lv, H. Kuang, G. Ma, J. Chen, and R. Li, Studies on ultrafast photocarrier recombination mechanisms of the Rh-doped $BaTiO_3$ photocatalyst, J. Phys. Chem. C **128**, 12239 (2024).

[32] J. Thompson, J. Nichols, S. Lee, S. Ryee, J. H. Gruenewald, J. G. Connell, M. Souri, J. M. Johnson, J. Hwang, M. J. Han, H. N. Lee, D.-W. Kim, and S. S. A. Seo, Enhanced metallic properties of $SrRuO_3$ thin films via kinetically controlled pulsed laser epitaxy, Appl. Phys. Lett. **109**, 161902 (2016).

[33] E. Chernova, O. Pacherova, D. Chvostova, A. Dejneka, T. Kocourek, M. Jelinek, and M. Tyunina, Strain-controlled optical absorption in epitaxial ferroelectric $BaTiO_3$ films, Appl. Phys. Lett. **106**, 192903 (2015).

[34] D. Pesquera, E. Parsonnet, A. Qualls, R. Xu, A. J. Gubser, J. Kim, Y. Jiang, G. Velarde, Y.-L. Huang, H. Y. Hwang, R. Ramesh, and L. W. Martin, Beyond substrates: strain engineering of ferroelectric membranes, Advanced Materials **32**, 2003780 (2020).

[35] M. Birkholz, *Thin Film Analysis by X-Ray Scattering* (Wiley-VCH, Weinheim, 2005).

[36] R. A. Young and D. B. Wiles, Profile shape functions in Rietveld refinements, J. Appl. Crystallogr. **15**, 430 (1982).

[37] B. J. Rodriguez, C. Callahan, S. V. Kalinin, and R. Proksch, Dual-frequency resonance-

tracking atomic force microscopy, Nanotechnology **18**, 475504 (2007).

[38] Y. Zhang, Y. Zhang, Q. Guo, X. Zhong, Y. Chu, H. Lu, G. Zhong, J. Jiang, C. Tan, M. Liao, Z. Lu, D. Zhang, J. Wang, J. Yuan, and Y. Zhou, Characterization of domain distributions by second harmonic generation in ferroelectrics, Npj Comput. Mater. **4**, 39 (2018).

[39] F. Löhl, V. Arsov, M. Felber, K. Hacker, W. Jalmuzna, B. Lorbeer, F. Ludwig, K.-H. Matthiesen, H. Schlarb, B. Schmidt, P. Schmüser, S. Schulz, J. Szewinski, A. Winter, and J. Zemella, Electron bunch timing with femtosecond precision in a superconducting free-electron laser, Phys. Rev. Lett. **104**, 144801 (2010).

[40] M. Czwalinna, R. Boll, H. Kirkwood, J. Koliyadu, J. Kral, B. Lautenschlager, R. Letrun, J. Liu, J. Müller, F. Pallas, D. Rivas, T. Sato, H. Schlarb, S. Schulz, and B. Steffen, Beam arrival stability at the European XFEL, Proceedings of the 12th International Particle Accelerator Conference **IPAC2021** (2021).

[41] R. Carley, B. Van Kuiken, L. Le Guyader, G. Mercurio, and A. Scherz, eds., *SCS Instrument Review Report* (European X-Ray Free-Electron Laser Facility GmbH, Schenefeld, 2022).

[42] E. A. Schneidmiller and M. V. Yurkov, Photon beam properties at the European XFEL (December 2010 revision), TESLA-FEL 2010-06 , 127 (2010).

[43] N. Gerasimova, D. La Civita, L. Samoylova, M. Vannoni, R. Villanueva, D. Hickin, R. Carley, R. Gort, B. E. Van Kuiken, P. Miedema, L. Le Guyarder, L. Mercadier, G. Mercurio, J. Schlappa, M. Teichman, A. Yaroslavtsev, H. Sinn, and A. Scherz, The soft X-ray monochromator at the SASE3 beamline of the European XFEL: from design to operation, J. Synchrotron Radiat. **29**, 1299 (2022).
