# Supplementary material for: Ultrafast decoupling of polarization and strain in ferroelectric BaTiO3
Source: Nat Commun. 2025 Aug 26;16:7966. doi: 10.1038/s41467-025-63045-6 (PMC12381263; doi:10.1038/s41467-025-63045-6)
Supplement: Supplementary file 2 — Reporting Summary [file 41467_2025_63045_MOESM2_ESM.pdf]

Corresponding author(s): Giuseppe Mercurio

Lasing Reporting Summary

Nature Research wishes to improve the reproducibility of the work that we publish. This form is intended for publication with all accepted papers reporting claims of lasing and provides structure for consistency and transparency in reporting. Some list items might not apply to an individual manuscript, but all fields must be completed for clarity.

For further information on Nature Research policies, including our data availability policy, see Authors & Referees.

Experimental design

Please check: are the following details reported in the manuscript?

1. Threshold

Plots of device output power versus pump power over a wide range of values indicating a clear threshold

☒ Yes ☐ No

Figure S10, Figure S11 show the reflected and SHG intensity as a function of laser pump power. Both reflectivity and SHG signals show a linear dependence on the input pump power. Figure S24 shows the SHG signal as a function of probe power, with a second order polynomial dependence on the incident probe power.

2. Linewidth narrowing

Plots of spectral power density for the emission at pump powers below, around, and above the lasing threshold, indicating a clear linewidth narrowing at threshold

☐ Yes ☒ No

In this study measurements of the spectral power density for the emission are not relevant, because we measure the reflected 800 nm beam or the SHG at 400 nm generated in the thin film.

Resolution of the spectrometer used to make spectral measurements

☐ Yes ☒ No

In this study, spectral measurements are not relevant because we focus on the total intensity, rather than the spectral properties.

3. Coherent emission

Measurements of the coherence and/or polarization of the emission

☒ Yes ☐ No

In SHG experiments, we measure either horizontal (p) or vertical polarization (s) of the 400 nm beam. These data are reported in Figure 2b-c and Figure S19.

4. Beam spatial profile

Image and/or measurement of the spatial shape and profile of the emission, showing a well-defined beam above threshold

☒ Yes ☐ No

Measurements of the beam spatial profile are reported the sections 'Time-resolved X-ray diffraction' and 'Time-resolved SHG and reflectivity' in Methods.

5. Operating conditions

Description of the laser and pumping conditions
Continuous-wave, pulsed, temperature of operation

☒ Yes ☐ No

A description of the laser pumping conditions is reported in the sections 'Time-resolved X-ray diffraction' and 'Time-resolved SHG and reflectivity' in Methods.

Threshold values provided as density values (e.g. W cm^{-2} or J cm^{-2}) taking into account the area of the device

☒ Yes ☐ No

Density values of the laser peak power intensity in W cm-2 are reported in the Abstract, in the section 'Photoinduced structural dynamics', in the section 'Discussion' and in Supplementary Note 4.

6. Alternative explanations

Reasoning as to why alternative explanations have been ruled out as responsible for the emission characteristics
e.g. amplified spontaneous, directional scattering; modification of fluorescence spectrum by the cavity

☒ Yes ☐ No

Figure S24 shows the SHG intensity as a function of the 800 nm probe pulse energy. The second order polynomial fit curve confirms the nonlinear nature of the signal measured by the photomultiplier. The reflectivity signal at 800 nm is filtered from the sum of 800 nm and 400 nm outgoing from the sample by means of a dichroic mirror.

7. Theoretical analysis

Theoretical analysis that ensures that the experimental values measured are realistic and reasonable
e.g. laser threshold, linewidth, cavity gain-loss, efficiency

☒ Yes ☐ No

The fit of SHG polar plots provides tensor elements with ratios in line with previous reports in literature, as reported in the section 'Fit curves of SHG data' in Methods.

8. Statistics

Number of devices fabricated and tested

☐ Yes ☒ No

We did not fabricate any device.

Statistical analysis of the device performance and lifetime (time to failure)

☐ Yes ☒ No

This information is not relevant because no devices were fabricated.
